# Food Safety Incident, Public Health Concern, and Risk Spillover Heterogeneity: Avian Influenza Shocks as Natural Experiments in China’s Consumer Markets

**DOI:** 10.3390/ijerph16214182

**Published:** 2019-10-29

**Authors:** Lan Yi, Jianping Tao, Zhongkun Zhu, Caifeng Tan, Le Qi

**Affiliations:** 1 College of Economics & Management, Huazhong Agricultural University, Wuhan 430070, China; yilan@webmail.hzau.edu.cn (L.Y.); zzk@mail.hzau.edu.cn (Z.Z.); tancaicai@webmail.hzau.edu.cn (C.T.); qile@webmail.hzau.edu.cn (L.Q.); 2 Hubei Rural Development Research Center, Wuhan 430070, China

**Keywords:** public health economics, price risk nonlinearity, price risk mechanism, price risk moderation, price risk mediation

## Abstract

*Background:* Food safety incidents have aroused widespread public health concern, causing food price risk. However, the causal paths remain largely unexplored in previous literature. This paper sets out to identify the relations of local and spatial spillovers of food safety incidents and public health concerns to food price risk in consumer markets within a setting with heterogeneous food safety risk levels. *Methods*: (i) Theoretically, unlike prior work, this paper decomposes food safety risks into food safety incidents (objective incident component) and public health concern (subjective concern component). This article develops a theoretical framework of causality to capture the underlying causal pathways motivated by the theories of limited attention and two-step flow of communication. (ii) Empirically, using avian influenza shocks in China’s poultry markets as natural experiments, this paper differentiates between low- and high-risk food and incidents. The article adopts dynamic spatial panel models to analyze potential nonlinearity, moderation, and mediation in the spillover of food safety risk to food price risk for a long panel of 30 provinces covering the November 2007 to November 2017 period. *Results:* (i) Food safety incident alone only triggers high-risk food price risk, not low-risk food price risk. (ii) Public health concern amplifies nonlinear food price risk triggered by food safety incident. (iii) High-risk incident intensifies negative pressure of public health concern on food price risk. (iv) Food safety incident indirectly affects high-risk food price risk through public health concern. *Conclusions:* Using a setting with heterogeneous risk levels, this paper documents that (i) food safety incident itself does not necessarily determine food price risk, whereas it is actually public health concern that directly causes nonlinear food price risk; (ii) public health concern spillover to food price risk is negatively moderated by high-risk incident, and (iii) food safety incident spillover to high-risk food price risk is mediated by public health concern. The findings complement current research by (i) elucidating the diverse impacts of food safety incident and public health concern on food price risk, which are obscure in previous literature, and (ii) highlighting that heterogeneous food and incident risk levels matter for determining food price risk spillover.

## 1. Introduction

In public health economics, food safety risk has become a major threat to the sustainability of global public health for both developed and developing countries in recent decades, causing 582 million occurrences of 22 diverse foodborne diseases (FBD), resulting in 351,000 fatalities since 2010 worldwide, according to World Health Organization (WHO) estimates from 2015 [1]. Food safety incidents, or events related to food safety risk, are recurrent outbreaks in the global consumer markets [2], including food fraud (FF) and its subcategory economically motivated adulteration (EMA), excessive use of certain inputs such as food additives owing to inadequate regulation or training, accidental food contamination due to negligence or unawareness, and naturally occurring hazards such as foot and mouth disease (FMD) and avian influenza (AI).

Public concern has focused more on health risk related to food safety, along with the economic development and risen incomes [3], and public health concern over food safety can become greater in the era of big data [4], where food safety incidents are increasingly covered by online news and social media [1], which have drawn increasing public attention to their roles by releasing and transmitting food safety incidents not only locally, but also globally [5], resulting in worldwide food scares [6].

Food prices in the global consumer markets fluctuate frequently and irregularly [7], leading to food price risk [8], and food safety incident is a key factor affecting food price risk [9]; moreover, public health concern over food safety intensified by online media coverage can enlarge the economic impacts of food safety incidents [10], exacerbating the food price risk associated with food safety risk [5]. For instance, an outbreak of animal infectious disease epidemics (such as bovine spongiform encephalopathy (BSE) or avian influenza (AI)) may arouse widespread public health concern over food safety and cause a food scare, resulting in livestock or poultry price risk. In terms of BSE shocks to the UK beef market during the 1990s, a 1% increase in the food publicity index led to a 1.70 pence/kg decrease in retail price, a 2.25 pence/kg decrease in wholesale price, and a 3.0 pence/kg decrease in producer price [11]; meanwhile, avian influenza shocks to China’s poultry markets in March 2013 decreased broiler chick price by 27% shortly after the outbreaks of the disease [12].

How exactly does food safety risk affect food price risk? The causal paths remain largely unexplored in previous literature. Understanding the paths of price risk spillover of food safety incident along with public health concern to the consumer markets in the big data era is important for explaining fundamental consumer decisions and economic impacts in the wake of increasingly rampant food scares, as well as for designing appropriate policy responses in public health economics.

In particular, this article seeks to address two main research questions:

**Q1:***Theoretically, how could the causal pathways be interpreted in terms of information communication?*
**Q2:***Empirically, how does food price risk spill over in a setting with heterogeneous food safety risk levels?*

In answering these research questions, our article contributes to the existing literature on the impact of food safety risk on consumer markets in public health economics by filling the knowledge gaps on theory and evidence.

First, theoretical gaps: (i) Unlike prior work where food safety risk is typically measured simply as a whole, we explicitly differentiate its potential components by decomposing food safety risk into food safety incident (objective incident component) and public health concern over food safety (subjective concern component), so as to calibrate the individual effects of its components rather than the total effects on food price risk. (ii) In contrast to previous research where food consumer decision was typically depicted by individual consumer risk perception models and media coverage of food safety, we allow for the clustering of consumer health concern, i.e., clustered public health concern, and use theories of limited attention and two-step flow of communication to interpret the social amplification of public health concern, which induces mass consumer herd behavior and subsequent drastic food price risk in the wake of extensive online media coverage of food scares in the age of big data. (iii) Differing from existing literature where the theoretical models are typically simplified by neglecting the mechanism through which food safety incident affects food price risk, we account for the amplifying effects of information and communication, laying out a theoretical framework of causality to unveil the detailed causal path of food price risk spillover of food safety incident, along with public health concern over food safety for the consumer markets in the era of big data.

Second, empirical gaps: (i) Unlike prior work, where time-series techniques based on a single district were typically implemented to analyze food price volatility and transmission, we assembled a long spatial panel dataset spanning 121 months and 30 provinces to conduct our empirical analysis, so as to address potential omitted variable concerns with respect to identifying the causal effects of food safety incident and public health concern on food price risk. (ii) In contrast to previous research where spatial analyses of food prices across markets have typically concentrated on food spatial market integration to test if the law of one price (LOP) holds, and in which empirical evidence on food price risk spatial spillover is exceedingly scarce especially for food price risk of animal products at the monthly level or higher levels, we have delved into the local and spatial spillovers of food safety incident and public health concern to food price risk, using exogenous avian influenza shocks to China’s poultry markets with monthly spatial panel data. (iii) Different from the existing literature where the effect of food safety risk on consumer markets is typically modeled by assuming that food scares as a whole could directly affect food price volatility and transmission without allowing for the underlying mechanism driving the effect, we implemented spatial analysis exploiting food price risk nonlinearity and food price risk mechanisms, including food price risk moderation and mediation. (iv) Unlike early work where empirical analyses were conducted in settings with homogeneous risk levels of food products and food safety incidents, and potential heterogeneity in causal effects with respect to different risk levels of food products and food safety incidents could not be fully captured, we have explicitly differentiated between low- and high-risk food and incidents, and further assessed food price risk spillover in a setting with heterogeneous food safety risk levels.

Note that, (i) “food safety risk” means the issue of food safety as a whole, which can be decomposed into food safety incident (objective incident component) and public health concern (subjective concern component); (ii) “food safety incident” means the objective incident component of “food safety risk”; (iii) “public health concern” means the subjective concern component of “food safety risk”; (iv) “food price risk spillover” means food safety risk spills over to the consumer markets and causes food price risk; (v) “on average” means the estimate in terms of the mean of the linear term of public health concern for a linear model; (vi) “in general” means the estimate in terms of the full distribution of the linear term of public health concern for a nonlinear model (i.e., the squared term) [5].

Overall, we set out to identify local and spatial spillovers of food safety incident and public health concern to food price risk in consumer markets, in settings with heterogeneous food safety risk levels, using big data techniques. More specifically, our main objectives are fourfold: in a setting with heterogeneous food safety risk levels, to test (i) whether food safety incident has negative local and spatial spillovers to high-risk food price risk; (ii) whether public health concern over food safety has nonlinear local and spatial spillovers to food price risk; (iii) whether expected moderation exists; that is, whether food safety incident negatively moderates the negative local and spatial spillovers of public health concern over food safety to food price risk; and (iv) whether expected mediation exists; that is, whether public health concern over food safety mediates the negative local and spatial spillovers of food safety incident to food price risk.

The remainder of the article is organized as follows: (i) Section 2 reviews related literature, formulating a theoretical framework and hypotheses; (ii) Section 3 defines data and variables, specifying empirical models; (iii) Section 4 presents our empirical findings; (iv) Section 5 discusses the relation to existing literature; and (v) Section 6 concludes.

We briefly summarize Section Introduction in Table 1.

## 2. Literature Review and Hypotheses

### 2.1. Related Literature

There has been a surge of recent interest in food safety in consumer markets, where we restrict our attention to the research on theory and evidence of the impact of food safety risk on consumer markets in public health economics:

First, theoretically, related literature focuses mainly on theory of food consumer decision, including food risk perception and media coverage of food safety. (i) Food risk perception. Lucht [13] points out that food risk and benefit perception, knowledge and trust, as well as personal value can affect consumer attitude. Zhou et al. [14] state that food scares and distrust in the government decreases food consumption and consumer willingness to pay (WTP); any food safety information exerts negative pressure on the consumer markets, while it makes no difference if the food safety information is risk perception decreasing, or if the information is risk perception increasing. (ii) Media coverage of food safety. Liu and Ma [1] argue that food risk perception might be nationwide instead of region specific, and consumers with more media exposure as well as higher education tend to be more concerned about food safety. Chen [15] suggests that consumer attention on food scandal news and food risk perception can influence consumer attitude.

Second, empirically, related literature focuses mainly on evidence of food price change, including food price volatility, food price transmission, and food price risk caused by food scare. (i) Food price volatility. Using a multiplicative MGARCH model, Serra [16] finds that producer food prices are positively correlated with consumer food prices, while geographical distance functions as a downward correction to the correlation, and long-run volatility dynamics are time-varying, while short-run volatility dynamics function as a downward correction to volatility correlation during quiet periods. Using the EEMD method, Wang et al. [17] decompose global food price volatility into low and high frequency components, finding that in the short run, the low-frequency component is determined by food policy and significant incident, while the high-frequency component is determined by routine adjustment and insignificant incident; in the long run, global food price volatility is determined by worldwide economic development. (ii) Food price transmission. Serra and Zilberman [18] conclude that in the long run, food price levels can be driven by energy prices, and the price risk in energy markets is also transmitted to food markets. Using petrol and maize prices, Dillon and Barrett [19] find that global oil prices transmit faster to local than to global food prices, indicating that the effects of commodity price shocks on local food prices are influenced mainly by transport costs, rather than by food prices, per se. (iii) Food price spatial analysis. Using a pairwise approach based on CPI (consumer price index) data for 153 goods, Iregui and Otero [20] find that food spatial market integration is more significant in unprocessed food products than in processed foods, other traded products, and nontraded products, and apart from nontraded products, the speed of food price differentials adjustment decreases with distance. Based on the international food trade network, Distefano et al. [21] analyze food spatial price dispersion and stochastic price distribution, finding that food spatial bilateral price dispersion is significant and continuous, indicating failure of the LOP; food price discrimination and food spatial price dispersion can be distinguished; and food price spikes and food price peaks are typically strongly correlated, leading to more severe food market fragmentation and food price discrimination in the wake of food price crises. (iv) Food price risk caused by food scare. Using a STAR model and avian influenza food scare information index based on the Egyptian poultry sector, Hassouneh et al. [22] find that poultry price adjustments are determined by food scare extent, and food scare causes wholesale margins to decrease, whereas food scare leads retailer margins to increase owing to retail market power. Using a RSVECM and an avian influenza information index based on the Turkish poultry market, Camoglu, Serra, and Gil [23] have found that producer prices respond to food scares slowly, whereas retail prices are responsive to the food scare extent.

### 2.2. Conceptual Framework of Decomposition

**Figure 1:**
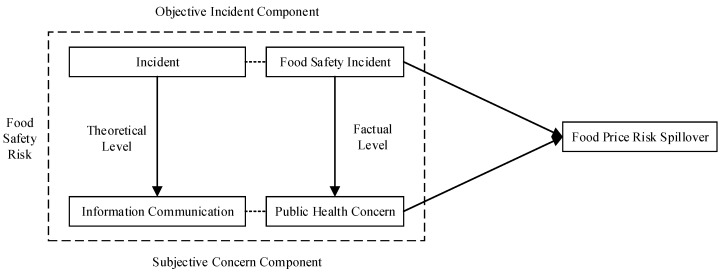
Conceptual framework of decomposition. *Source:* Originally developed by the authors.

Unlike prior work where food safety risk is typically theoretically measured simply as a whole, we explicitly differentiate its potential components by developing a conceptual framework of decomposition, which decomposes food safety risk into an objective incident component (theoretical level as “incident”, versus factual level as “food safety incident”) and subjective concern component (theoretical level as “information communication”, versus factual level as “public health concern”), so as to calibrate the individual effects of its components, rather than the total effects, on food price risk. Following the methodology of Hong and Stein [24] and Li et al. [25], we use the theory of limited attention [26] and the theory of two-step flow of communication [27] to delve into food price risk spillover of food safety risk to consumer markets, from the perspective of public health concern over food safety. We illustrate our conceptual framework of decomposition in Figure 1.

### 2.3. Theoretical Framework of Causality

**Figure 2:**
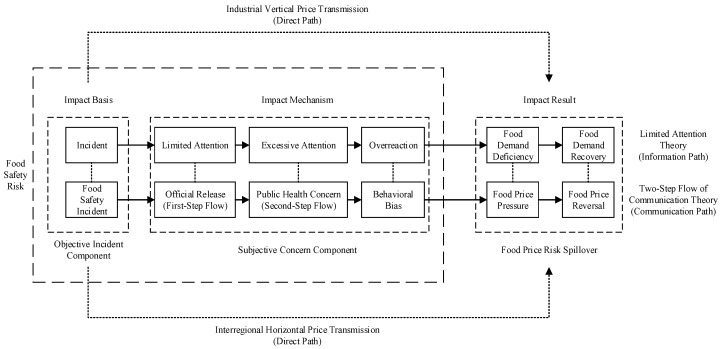
Theoretical framework of causality. *Source:* Originally developed by the authors.

Accounting for the influence of online media on public health concern, by combining the information and communication paths, we associate limited attention of market to source information, with overreaction of the market to information amplified by online media’s second-step flow, and develop a theoretical framework of causality to capture causal effects of food safety incident and public health concern on food price risk, showing that (i) source information on food safety incidents, which may only receive limited consumers’ attention, is initially released by the authorities during first-step flow; (ii) source information is amplified by increased public health concern stemming from substantial online media coverage during second-step flow, leading to excessive consumer attention; (iii) clustered public health concern can give rise to mass consumer behavioral biases, triggering market overreaction to the incident information; (iv) in the short run, the deficiency of food market demand can give rise to food price pressure; and (v) in the long run, the recovery of food market demand can result in food price reversal. We illustrate our theoretical framework of causality in Figure 2.

Rather than follow classical models in simply assuming that food safety risk as a whole affects food price risk through two direct paths, i.e., industrial vertical price transmission (namely, food prices throughout supply chain) and interregional horizontal price transmission (namely, spatial correlation in food prices); we propose two novel indirect paths, i.e., information path (motivated by the theory of limited attention [26]) and communication path (motivated by the theory of two-step flow of communication [27]): (i) information path; namely, incident → limited attention → excessive attention → overreaction → food demand deficiency → food demand recovery; and (ii) communication path; namely, food safety incident → official release (first-step flow) → public health concern (second-step flow) → behavioral bias → food price pressure → food price reversal.

By integrating the information path and communication path, we cautiously interpret our theoretical framework of causality with respect to food safety incident, public health concern, and food price risk spillover: (i) Limited attention to source information on food safety incident, namely, food safety incident (incident) → official release (first-step flow) (limited attention). In the big data era, consumers may be immediately informed of food safety incidents (such as avian influenza, bovine spongiform encephalopathy, and melamine) through official release, which is the first-step flow of source information on food safety incident, where consumers might have incomplete information about food markets; hence, the first-step flow of source information can draw limited attention of consumers [28]. (ii) Excessive attention to incident information amplified by public health concern, namely, initial release (first-step flow) (limited attention) → public health concern (second-step flow) (excessive attention). After being officially released, source information flows from the authorities to online news and social media (e.g., Weibo, WeChat, Facebook, and Twitter) who serve as online opinion leaders, and in turn from online opinion leaders to inactive individuals [27]; online media disseminate the information on food safety incident during second-step flow, which leads to a surge of online media coverage, arousing widespread public health concern over food safety; under the influence of public health concern over food safety, consumers’ risk perceptions of food safety heighten, drawing excessive attention. (iii) Bounded rationality and behavioral bias, namely, public health concern (second-step flow) (excessive attention) → behavioral bias (overreaction). Due to consumers’ asymmetric information on food markets and uncertain expectations of future food prices, consumers’ bounded rationality can lead to behavioral biases in food product purchase decisions, and herding behavior can give rise to widespread consumer panic and food scares, which in turn can result in food market overreaction. (iv) Overreaction and food price risk, namely, behavioral bias (overreaction) → food price pressure (food demand deficiency). Food market overreaction can lead to a critical deficiency in effective food demand, which in turn directly causes a collapse of food prices [29]. (v) Food price reversal after food safety incident, namely, food price pressure (food demand deficiency) → food price reversal (food demand recovery). After the magnitude of public health concern over food safety goes down, consumer demand for food products recovers, which in turn gives rise to the reversal of food markets, and then food prices gradually recover to pre-incident levels [30].

Moreover, there may exist potential positive feedback mechanisms in the causal paths: (i) Excessive attention to incident information may lead to a significant reduction of food consumption (overreaction), which in turn can give rise to food demand deficiency and negative food price pressure, causing food price risk. (ii) Conversely, related food price risk may draw more consumer attention, and more consumer attention intensifies consumer overreaction, which in turn aggravates food price pressure, causing higher food price risk. (iii) Altogether, causality can run from attention to price risk, and can also run from price risk to attention: excessive attention → overreaction → food price risk → more attention → intensified overreaction → higher food price risk → even more attention.

### 2.4. Theoretical Hypotheses

Using the theory of limited attention [26] and the theory of two-step flow of communication [27], we formulate our theoretical hypotheses based on conceptual framework of decomposition (Figure 1) and theoretical framework of causality (Figure 2).

(i) Heterogeneous food price risk nonlinearity. In the big data era, food safety incidents arouse widespread public health concern, causing heterogeneous food price risk spillover locally and spatially. We explicitly differentiate the potential components of food safety risk by decomposing food safety risk into food safety incident (objective incident component) and public health concern over food safety (subjective concern component), so as to calibrate the individual effects of its components, rather than the total effects, on food price risk. We assume that market prices of heterogeneous food may respond differently to the same realizations of the shocks, and therefore formulate theoretical hypotheses H1–H2.

**H1:***Food safety incident has negative local and spatial spillovers to food price risk, which is heterogeneous in low- and high-risk food.*
**H2:***Public health concern over food safety has nonlinear local and spatial spillovers to food price risk, which is heterogeneous in low- and high-risk food: (i) on average, public health concern over food safety has negative local and spatial spillovers to food price risk, which is heterogeneous in low- and high-risk food; and (ii) in general, public health concern over food safety has inverse U-shaped local and spatial spillovers to food price risk, which is heterogeneous in low- and high-risk food.*

(ii) Heterogeneous food price risk mechanism. We account for the amplifying effects of information and communication, and allow for the potential impact mechanism through which food safety incident affects food price risk, so as to unveil the detailed causal path of food price risk spillover of food safety incident along with public health concern over food safety to the consumer markets in the big data era. We assume that the causal path may be heterogeneous in terms of diverse food and incidents, and therefore formulate theoretical hypotheses H3–H4.

**H3:***Food safety incident negatively moderates the negative local and spatial spillovers of public health concern over food safety to food price risk, which is heterogeneous in low- and high-risk food and incidents.*
**H4:***Public health concern over food safety mediates the negative local and spatial spillovers of food safety incident to food price risk, which is heterogeneous in low- and high-risk food and incidents.*

**Figure 3:**
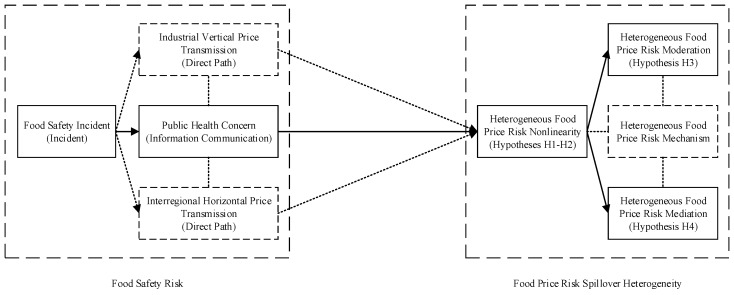
Analytical framework of heterogeneity. *Source:* Originally developed by the authors.

We therefore propose an analytical framework of heterogeneity (Figure 3) to facilitate the analysis.

## 3. Materials and Methods

### 3.1. Research Design: Avian Influenza Shocks as Natural Experiments

There exist diverse types of food safety risk in consumer markets. Some previous empirical studies use evidence from food safety scandals such as food fraud or economically motivated adulteration (FF/EMA), which may suffer from reverse causality. They are as follows: (i) an increase in the number of food safety scandals can lead to a decline in consumer confidence and related food demand, i.e., causality can run from supply to demand; (ii) food market environments could also influence outbreaks of food safety scandals, i.e., causality might also run from demand to supply; (iii) taken together, incidence of food safety scandals might be endogenous to the food market response.

We adopt a natural experiment approach to address this issue. Given that an outbreak of animal infectious disease epidemics (such as avian influenza and bovine spongiform encephalopathy), which may arouse widespread public health concern over food safety and cause a related food scare, is an exogenous shock that happens spontaneously and stochastically and does not suffer from reverse causality, thus providing us with ideal natural experiments for causal inference, we employ avian influenza epidemic to represent food safety risk, and adopt a natural experiment approach by using the dynamic impact of exogenous avian influenza shocks on China’s poultry markets to correctly identify the causal effect. Using avian influenza shocks to consumer markets as natural experiments, we resolve this endogeneity concern of previous related empirical studies, and thereby correctly identify the causal effect of food safety risk on food price risk.

### 3.2. Data

Our data sample consists of China’s 30 provinces (Tibet, Hong Kong, Macao, and Taiwan are not included because of insufficient administrative data), spanning November 2007 to November 2017.

We use 5 types of monthly provincial panel data to delve into the spillover of food safety risk to food price risk: (i) food price data, obtained from China Animal Agriculture Association (CAAA) (http://www.caaa.cn); (ii) search engine data, hand-collected from Baidu Search (https://www.baidu.com) and Google search (https://www.google.com); (iii) official journal data, hand-collected from Official Veterinary Bulletin (http://www.moa.gov.cn/gk/sygb/) and Disease Surveillance (http://www.jbjc.org/indexen.htm); (iv) food supply and demand data, obtained from EPS China Data (http://www.epschinadata.com); and (v) shapefile (map), obtained from GADM data (https://www.gadm.org/download_country_v3.html).

### 3.3. Variables

#### 3.3.1. Dependent Variables: Food Price Risk

On measuring food price risk, we further differentiate between low-risk food (as measured by dressed broiler price) and high-risk food (as measured by live broiler price), assuming that food price risks of low- and high-risk food respond differently to the same realizations of exogenous food safety shocks.

The reason why we use dressed broiler price to measure low-risk food price risk, and use live broiler price to measure high-risk food price risk, is that (i) dressed broiler product, which is located in the downstream food industrial chain, could be safer to consume, due to mass production stricter food market regulation, rendering it relatively low-risk; and (ii) live broiler product, which is located in the midstream food industrial chain, might be more risky to consume than its counterpart, since live poultry infected with H5 subtype of highly pathogenic avian influenza (HPAI) and H7N9 can be contagious and lead to foodborne disease (FBD), rendering it relatively high-risk.

#### 3.3.2. Key Independent Variables: Food Safety Incident and Public Health Concern

(i) Food safety incident.

On measuring food safety incident (objective incident component of food safety risk), we further differentiate between low-risk incident (as measured by poultry infection with avian influenza incident dummy) and high-risk incident (as measured by human infection with avian influenza incident dummy), and we hand-collect province-level panel data on low- and high-risk incidents from two Chinese official journals (i.e., Official Veterinary Bulletin and Disease Surveillance), respectively.

The reason why we use poultry infection with an avian influenza incident dummy to measure low-risk food safety incident, and use human infection with avian influenza incident dummy to measure high-risk food safety incident is that (i) poultry infection with avian influenza incident, which can be classified as nonhuman-related, could be much less influential in affecting consumers’ food risk perceptions, due to the fact that the lack of direct evidence on the confirmed human cases is less likely to draw consumers’ attention, which can result in limited attention to poultry infection incident in consumer markets, rendering it relatively low-risk; and (ii) human infection with avian influenza incident, which can be classified as human-related, might exert a substantial impact on poultry markets, since the reported human cases can become a salient event, and which may dramatically elevate consumers’ food risk perceptions, resulting in excessive attention and overreaction to human infection incident in consumer markets, rendering it relatively high-risk.

The approach we take to measure low-risk food safety incident and high-risk food safety incident is conducted as follows: (i) we hand-collect monthly provincial administrative panel data on poultry infection with avian influenza cases from Official Veterinary Bulletin, the official journal of Ministry of Agriculture and Rural Affairs of China, and hand-collect monthly provincial administrative panel data on human infection with avian influenza cases from Disease Surveillance, the official journal of China’s Centre for Disease Control and Prevention; (ii) we transform these hand-collected continuous variables into dummies, indicating whether poultry infection with avian influenza incident or human infection with avian influenza incident outbreaks, respectively; and (iii) we assemble these dummies and construct a monthly provincial panel of low-risk food safety incident and high-risk food safety incident.

For robustness checks, we measure low-risk food safety incident using poultry infection with avian influenza cases, and measure high-risk food safety incident using human infection with avian influenza cases, both of which are the original hand-collected continuous variables taken from the two official journals.

(ii) Public health concern over food safety.

On measuring public health concern over food safety (subjective concern component of food safety risk), we use public health concern over avian influenza (as measured by Baidu search volume on avian influenza) to represent public health concern over food safety, and we hand-collect province-level panel data on public health concern over food safety from the Baidu search volume; we also include the squared term of public health concern over food safety to capture potential price risk nonlinearity.

The reason why we use the Baidu search volume on avian influenza to measure public health concern over food safety is that (i) Baidu Search, a search engine giant, which has the largest search engine in China, can link to more media reports and provide more detailed information on avian influenza across provinces; (ii) Baidu Search was founded at the beginning of 2000, which can meet the requirements for our data sample spanning November 2007–November 2017; (iii) Baidu Search can be used to search for keywords for a customized time period (e.g., one month in this article), which enables us to construct a monthly provincial panel of public health concern over food safety; and (iv) Baidu search volume can comprehensively represent attention of online media and consumers to food safety; that is, public health concern over food safety.

The approach we take to measure public health concern over food safety is conducted as follows: (i) we perform a search on Baidu Search using the keywords “avian influenza” and “Beijing” (in Chinese); (ii) we restrict the search results to the November 2007 period, and record the number of results; (iii) we continue to search for “avian influenza” and “Beijing” for the period November 2008, …, November 2017, and record the number of results, respectively; and (iv) we repeat this procedure using the keywords “avian influenza” and “Tianjin”, …, “avian influenza” and “Xinjiang”, altogether 30 provinces for 121 months, constructing a monthly provincial panel of public health concern over food safety.

For robustness checks, we measure public health concern over food safety using Google search volume on avian influenza, which is hand-collected from Google search using a similar approach as the Baidu Search approach.

#### 3.3.3. Control Variables

(i) Price control variables: industrial vertical price transmission.

We control for industrial vertical price transmission (namely, food prices throughout supply chain) by including a number of food price control variables (that is, broiler feed price, broiler chick price, live broiler price, dressed broiler price, and pork price); where broiler feed price and broiler chick price are controls for raw material prices in the upstream food industrial chain, live broiler price is a control for intermediate product price in the midstream food industrial chain, dressed broiler price is a control for consumer product price in the downstream food industrial chain, and pork price is a control for poultry substitute price.

(ii) Supply and demand control variables: market supply and demand.

We control for food market supply and demand by including several food supply and demand control variables (that is, aggregate poultry output, urban poultry consumption, and rural poultry consumption); where aggregate poultry output is a control for food market supply, and urban poultry consumption and rural poultry consumption are controls for food market demand.

#### 3.3.4. Spatial Weighting Matrices: Interregional Horizontal Price Transmission

We use the spatial weighting matrix to characterize the spatial relationships between areas; that is, the potential spatial spillover of food safety risk to food price risk, thereby capturing interregional horizontal price transmission (namely, spatial correlation in food prices). We collect the shapefile (map) for Chinese provinces from GADM data, and construct the spatial weighting matrices based on this shapefile, using the spshape2dta and spwmatrix commands in Stata.

(i) Squared inverse-distance spatial weighting matrix.

Our main spatial weighting matrix is the squared inverse-distance spatial weighting matrix [31], where (i, j) element = 1/d^2^ if i ≠ j, and (i, j) element = 0 if i = j; where d refers to the geographical distance between the centroids of provinces i and j, and the matrix is row-standardized.

(1) $$W(1):w_{{}_{ij}}^{s}=\left\{ \begin{array}{l} \frac{1}{d_{ij}{}^{2}},i\neq j\\ 0,i=j \end{array}\right.\;w_{{}_{ij}}^{\prime s}=\left\{ \begin{array}{l} \frac{w_{{}_{ij}}^{s}}{\sum_{j=1}^{n}w_{{}_{ij}}^{s}},i\neq j\\ 0,i=j \end{array}\right.$$

(ii) Exponential inverse-distance spatial weighting matrix.

We use the exponential inverse-distance spatial weighting matrix [32] in robustness checks, where (i, j) element = $1/e^{d/d_{\min}}$ if i ≠ j, and (i, j) element = 0 if i = j; where d refers to the geographical distance between the centroids of provinces i and j, and the matrix is row-standardized.

(2) $$W(2):w_{{}_{ij}}^{e}=\left\{ \begin{array}{l} \frac{1}{e^{d_{ij}/d_{\min}}},i\neq j\\ 0,i=j \end{array}\right.\;w_{{}_{ij}}^{\prime e}=\left\{ \begin{array}{l} \frac{w_{{}_{ij}}^{e}}{\sum_{j=1}^{n}w_{{}_{ij}}^{e}},i\neq j\\ 0,i=j \end{array}\right.$$

We summarize the aforementioned variable measurements and data sources in Table 2.

### 3.4. Research Methods

To answer the research questions raised in Section Introduction, we adopt theoretical and empirical methods.

Theoretical methods: Unlike prior work, where food safety risk is usually measured simply as a whole, we establish a conceptual framework of decomposition, decomposing food safety risk into food safety incident (objective incident component) and public health concern over food safety (subjective concern component), so as to discriminate between incident and information communication; furthermore, following Hong and Stein [24], Li et al. [25], and Lan et al. [5], we arrange a theoretical framework of causality for assessing the causal effects of food safety incident, public health concern, and food price risk, motivated by the theory of limited attention [26] and the theory of two-step flow of communication [27].

Empirical methods: In order to address the endogeneity concerns of previous related studies and thereby correctly identify the causal effects, we adopt a natural experiment approach by using the dynamic impact of exogenous avian influenza shocks on China’s poultry markets, for a panel of 30 provinces spanning November 2007–November 2017, with big data techniques; following our previous work [5], by establishing an theoretical framework and differentiating between low- and high-risk food and incidents, we assess the food price risk spillover heterogeneity: (i) we develop the benchmark spatial models to analyze the heterogeneous food price risk nonlinearity; and (ii) we extend the benchmark spatial models by allowing for interaction effects to capture the heterogeneous food price risk moderation, and by applying the causal steps approach [33] to capture the heterogeneous food price risk mediation.

#### 3.4.1. Empirical Strategy

Following our analytical framework of heterogeneity (Figure 3), to test hypotheses H1–H4, we implement the following procedures:

First, to test heterogeneous food price risk nonlinearity (hypotheses H1–H2): (i) we developed a nonspatial baseline model which includes squared public health concern and two-way FE, to characterize the potential nonlinearity in the relationship between food price risk and public health concern. (ii) We performed the Moran test for potential spatial correlation in food price risk. (iii) As food price risk is spatially correlated, we extended the nonspatial baseline model to incorporate temporal, spatiotemporal, and spatial variables, so that we develop a series of static and dynamic spatial panel models (including SDM, dynamic SDM, SAR, dynamic SAR, SAC, and SEM), to characterize the nonlinear local and spatial spillovers of food safety incident and public health concern to food price risk. (iv) We conducted a formal spatial model selection exercise to find a spatial model which best fits the data. (v) As dynamic SAR best fits the data, and dynamic SDM fits second-best, we selected dynamic SAR as our main spatial model, and dynamic SDM as an alternative. (vi) We estimated dynamic SAR and dynamic SDM, with low- and high-risk food price risk as dependent variables, respectively. (vii) Using spatial coefficients (ρ), we tested whether spatially lagged effects of food price risk should be accounted for; and using BIC, we tested whether dynamic SAR better fits the data than dynamic SDM. (viii) As all spatial coefficients are significant, and dynamic SAR better fits the data, we examined the short- and long-run local and spatial spillovers of low-risk incident, high-risk incident, public health concern, and squared public health concern, respectively, to test if hypotheses H1–H2 held. (ix) As squared public health concern is significantly negative, we further computed the turning point values for public health concern in the inverse U-shapes.

Second, to test heterogeneous food price risk moderation (hypothesis H3): (i) Step 1, as dynamic SAR best fits the data, we estimated a simplified dynamic SAR, where we only included low-risk incident and public health concern, while high-risk incident and squared public health concern are not included, with low- and high-risk food price risk as dependent variables, respectively. (ii) Step 2, we estimated an extended dynamic SAR, where we allow for the interaction between low-risk incident and public health concern, with low- and high-risk food price risk as dependent variables, respectively. (iii) We repeated Steps 1–2, replacing low-risk incident with high-risk incident. (iv) Using spatial coefficients (ρ), we tested whether spatially lagged effects of food price risk should be accounted for. (v) As all spatial coefficients are significant, we examined the short- and long-run local and spatial spillovers of the interaction between low-risk incident and public health concern, and the interaction between high-risk incident and public health concern, respectively, to test if hypothesis H3 would hold.

Third, to test heterogeneous food price risk mediation (hypothesis H4). (i) Step 1, as dynamic SAR best fits the data, we estimated a simplified dynamic SAR where we regressed food price risk on low-risk incident, with low- and high-risk food price risk as dependent variables, respectively, to test whether low-risk incident is significant: if low-risk incident is significant, we turned to Step 2; and if low-risk incident is insignificant, we ended mediation analysis. (ii) Step 2, we estimated another simplified dynamic SAR, where we regress public health concern on low-risk incident, with low- and high-risk food price risk as dependent variables, respectively, to test whether low-risk incident is significant: if low-risk incident was significant, we turned to Step 3; and if low-risk incident was insignificant, we conducted the Sobel test for the specific mediation effect. (iii) Step 3, we estimated an extended dynamic SAR where we regress food price risk on low-risk incident and public health concern, with low- and high-risk food price risk as dependent variables, respectively, to test whether low-risk incident and public health concern were significant: First, if public health concern was significant, mediation exists; and if public health concern was insignificant, we conducted the Sobel test for the specific mediation effect. Second, if low-risk incident was significant, partial mediation exists; and if low-risk incident was insignificant, complete mediation exists. (iv) We repeated Steps 1–3, replacing low-risk incident with high-risk incident. (v) Using spatial coefficients (ρ), we tested whether spatially lagged effects of food price risk should be accounted for. (vi) As all spatial coefficients are significant, we examined the short- and long-run local and spatial spillovers of the key variables mentioned in Steps 1–3, respectively, to test if hypothesis H4 held. (vii) As public health concern mediates the spillovers of low- and high-risk incident to high-risk food price risk, we further computed the related mediation effect (ME) and ratio of mediation effect to total effect (MR).

#### 3.4.2. Specifications of Heterogeneous Food Price Risk Nonlinearity: Hypotheses H1–H2

Since dynamic spatial models can characterize both short- and long-run local and spatial spillovers, we developed dynamic SDM and dynamic SAR models [34] to test for potential heterogeneous food price risk nonlinearity. Dynamic SDM is represented in Equation (3):

(3) $$\begin{array}{l} \ln lrfp_{it}=\ln\alpha +\tau \ln lrfp_{it-1}+\psi \sum_{j=1}^{n}w_{ij}\ln lrfp_{jt-1}+\rho \sum_{j=1}^{n}w_{ij}\ln lrfp_{jt}\\ +\beta_{1}lrid_{it}+\beta_{2}hrid_{it}+\beta_{3}\ln phcb_{it}+\beta_{4}\ln phcb2_{it}+\beta_{5}X_{it}\\ +\theta_{1}\sum_{j=1}^{n}w_{ij}lrid_{it}+\theta_{2}\sum_{j=1}^{n}w_{ij}hrid_{it}+\theta_{3}\sum_{j=1}^{n}w_{ij}\ln phcb_{it}+\theta_{4}\sum_{j=1}^{n}w_{ij}\ln phcb2_{it}+\gamma month_{t}+\mu_{i}+\varepsilon_{it} \end{array}$$

where τ, ψ, and ρ are a temporally lagged effect, spatiotemporally lagged effect, and spatially lagged effects of dependent variable, respectively; β and θ are main coefficients and spatially lagged effects of key independent variables, respectively; and γ and μ are month fixed effects (linear monthly trend) and province fixed effects (province dummies), respectively. A special case of dynamic SDM is dynamic SAR, which is represented in Equation (4):

(4) $$\begin{array}{l} \ln lrfp_{it}=\ln\alpha +\tau \ln lrfp_{it-1}+\psi \sum_{j=1}^{n}w_{ij}\ln lrfp_{jt-1}+\rho \sum_{j=1}^{n}w_{ij}\ln lrfp_{jt}\\ +\beta_{1}lrid_{it}+\beta_{2}hrid_{it}+\beta_{3}\ln phcb_{it}+\beta_{4}\ln phcb2_{it}+\beta_{5}X_{it}+\gamma month_{t}+\mu_{i}+\varepsilon_{it} \end{array}$$

where spatially lagged effects of key independent variables (θ) are not included, compared with dynamic SDM.

#### 3.4.3. Specifications of heterogeneous food price risk moderation: hypothesis H3

Step 1: We begin with a simple dynamic SAR adapted from Equation (4) to see whether low-risk incident and public health concern local and spatial spillover to low-risk food price risk, respectively:

(5) $$\begin{array}{l} \ln lrfp_{it}=\ln\alpha +\tau \ln lrfp_{it-1}+\psi \sum_{j=1}^{n}w_{ij}\ln lrfp_{jt-1}+\rho \sum_{j=1}^{n}w_{ij}\ln lrfp_{jt}\\ +\beta_{2}lrid_{it}+\beta_{1}\ln phcb_{it}+\beta_{3}X_{it}+\gamma month_{t}+\mu_{i}+\varepsilon_{it} \end{array}$$

where we only include low-risk incident (lrid) and public health concern (lnphcb), while high-risk incident (hrid) and squared public health concern (lnphcb2) are not included, compared with Equation (4).

Step 2: We extend Equation (4) by allowing for the interaction between low-risk incident and public health concern, to see whether low-risk incident and public health concern interact in their local and spatial spillovers to low-risk food price risk:

(6) $$\begin{array}{l} \ln lrfp_{it}=\ln\alpha +\tau \ln lrfp_{it-1}+\psi \sum_{j=1}^{n}w_{ij}\ln lrfp_{jt-1}+\rho \sum_{j=1}^{n}w_{ij}\ln lrfp_{jt}\\ +\beta_{1}lrid_{it}+\beta_{2}\ln phcb_{it}+\beta_{3}lrid_{it}\times \ln phcb_{it}+\beta_{4}X_{it}+\gamma month_{t}+\mu_{i}+\varepsilon_{it} \end{array}$$

where the interaction between low-risk incident and public health concern (lrid × lnphcb) is included, compared with Equation (5).

#### 3.4.4. Specifications of Heterogeneous Food Price Risk Mediation: Hypothesis H4

Following the causal steps approach in Baron and Kenny [33], we started with three simple conceptual models for mediation analysis:

For our analysis of heterogeneous food price risk mediation, we extended the causal steps approach developed in Baron and Kenny [33] to an extended spatial causal steps approach, by incorporating temporally lagged effect (τ), spatiotemporally lagged effect (ψ), and spatially lagged effect (ρ) of low-risk food price risk, to see whether public health concern mediates the local and spatial spillovers of low-risk incident to low-risk food price risk:

Step 1: We begin with a simple dynamic SAR, adapted from Equation (4), and regress low-risk food price risk on low-risk incident, to test whether β_1_ in Equation (7) is significant:

(7) $$\begin{array}{l} \ln lrfp_{it}=\ln\alpha +\tau \ln lrfp_{it-1}+\psi \sum_{j=1}^{n}w_{ij}\ln lrfp_{jt-1}+\rho \sum_{j=1}^{n}w_{ij}\ln lrfp_{jt}\\ +\beta_{1}lrid_{it}+\beta_{2}X_{it}+\gamma month_{t}+\mu_{i}+\varepsilon_{it} \end{array}$$

Step 2: We regress public health concern on low-risk incident, to test whether β_1_ in Equation (8) is significant:

(8) $$\begin{array}{l} \ln phcb_{it}=\ln\alpha +\tau \ln phcb_{it-1}+\psi \sum_{j=1}^{n}w_{ij}\ln phcb_{jt-1}+\rho \sum_{j=1}^{n}w_{ij}\ln phcb_{jt}\\ +\beta_{1}lrid_{it}+\beta_{2}X_{it}+\gamma month_{t}+\mu_{i}+\varepsilon_{it} \end{array}$$

Step 3: We regress low-risk food price risk on low-risk incident and public health concern, to test whether β_1_ and β_2_ in Equation (9) are significant:

(9) $$\begin{array}{l} \ln lrfp_{it}=\ln\alpha +\tau \ln lrfp_{it-1}+\psi \sum_{j=1}^{n}w_{ij}\ln lrfp_{jt-1}+\rho \sum_{j=1}^{n}w_{ij}\ln lrfp_{jt}\\ +\beta_{1}lrid_{it}+\beta_{2}\ln phcb_{it}+\beta_{3}X_{it}+\gamma month_{t}+\mu_{i}+\varepsilon_{it} \end{array}$$

## 4. Results

### 4.1. Summary Statistics

Our original data sample may suffer from several issues: (i) due to diverse magnitude, dimensions, and units, the relative importance of low-risk incident, high-risk incident, and public health concern in predicting food price risk may not be easily assessed; (ii) the standard deviation, skewness, and excess kurtosis are relatively high; (iii) potential heteroskedasticity, outliers, and multicollinearity may affect our estimates.

To address these concerns, (i) we used multiple imputation to impute missing data; (ii) all monetary values are deflated to January 2004 prices using the CPI; (iii) for all continuous variables, we take logarithms to deal with heteroskedasticity, winsorize each variable at top and bottom 1 percent to deal with outliers, and standardize each variable at the province level to be mean 0 and standard deviation 1 to alleviate multicollinearity concerns and facilitate interpretation; and (iv) for lnphcb (public health concern), lnlric (poultry infection case), and lnhric (human infection case), we replaced lny with ln(1+y) where the variable y is nonnegative but can take on the value 0 [35]. Table 3 reports summary statistics for our main variables after being processed.

### 4.2. Benchmark Analysis of Heterogeneous Food Price Risk Nonlinearity: Hypotheses H1–H2

By allowing for nonlinearity and spatial effects by including squared public opinion and spatial variables, we sought to test whether food safety incident has negative local and spatial spillovers to high-risk food price risk, which is heterogeneous in low- and high-risk food (hypothesis H1); and whether public health concern over food safety has nonlinear local and spatial spillovers to food price risk, which is heterogeneous in low- and high-risk food (hypothesis H2). We estimated Equation (3) (dynamic SDM, the second-best fit model) and Equation (4) (dynamic SAR, the first-best fit model) using QML estimator, excluding and including squared public health concern (lnphcb2), respectively, all with FE, as reported in Table 4: (i) columns (1)–(4) report estimates of Equation (3) (dynamic SDM) excluding lnphcb2, Equation (3) (dynamic SDM) including lnphcb2, Equation (4) (dynamic SAR) excluding lnphcb2, and Equation (4) (dynamic SAR) including lnphcb2, respectively, with lnlrfp (low-risk food price risk) as DV (dependent variable); and (ii) columns (5)–(8) report estimates with lnhrfp (high-risk food price risk) as DV.

First, spatial specification diagnostic tests: (i) using spatial coefficients (ρ), we find that all columns are statistically significant, suggesting that spatially lagged effects of food price risk should be accounted for. (ii) using BIC, we find that dynamic SAR best fits the data; therefore, we restrict attention to dynamic SAR (columns (3)–(4) and (7)–(8)).

Second, empirical analysis for low-risk food: (columns (3)–(4)). (i) In columns (3)–(4), the short-run local spillovers (SR_Direct_lrid and SR_Direct_hrid), short-run spatial spillovers (SR_Indirect_lrid and SR_Indirect_hrid), long-run local spillovers (LR_Direct_lrid and LR_Direct_hrid), and long-run spatial spillovers (LR_Indirect_lrid and LR_Indirect_hrid) of low-risk incident and high-risk incident are all statistically insignificant, suggesting no significant local or spatial spillover of food safety incident. Thus, whether in the short run or in the long run, neither low-risk incident nor high-risk incident has a significant local or spatial spillover to low-risk food price risk.

(ii) First, in column (3), the short-run local spillover (SR_Direct_lnphcb) and short-run spatial spillover (SR_Indirect_lnphcb) of public health concern are both negative, suggesting negative local and spatial spillovers of public health concern on average; second, in column (4), the short-run local spillover (SR_Direct_lnphcb2) and short-run spatial spillover (SR_Indirect_lnphcb2) of squared public health concern are both negative, suggesting inverse U-shaped local and spatial spillovers of public health concern in general. Thus, in the short run, on average, public health concern over food safety has negative local and spatial spillovers to low-risk food price risk; in general, public health concern over food safety has inverse U-shaped local and spatial spillovers to low-risk food price risk.

(iii) First, in column (3), the long-run local spillover (LR_Direct_lnphcb) of public health concern is negative, and long-run spatial spillover (LR_Indirect_lnphcb) of public health concern is statistically insignificant, suggesting a negative local spillover but no significant spatial spillover on average; second, in column (4), the long-run local spillover (LR_Direct_lnphcb2) of squared public health concern is negative, and long-run spatial spillover (LR_Indirect_lnphcb2) of squared public health concern is statistically insignificant, suggesting an inverse U-shaped local spillover but no significant spatial spillover in general. Thus, in the long run, public health concern over food safety has a negative local spillover to low-risk food price risk on average, and an inverse U-shaped local spillover to low-risk food price risk in general; public health concern over food safety has no significant spatial spillover to low-risk food price risk.

Third, empirical analysis for high-risk food: (columns (7)–(8)). (i) First, in columns (7)–(8), the short-run local spillovers (SR_Direct_lrid and SR_Direct_hrid) and short-run spatial spillovers (SR_Indirect_lrid and SR_Indirect_hrid) are all negative, suggesting negative local and spatial spillovers of food safety incident; second, in column (7), the short-run local spillover (SR_Direct_lnphcb) and short-run spatial spillover (SR_Indirect_lnphcb) of public health concern are both negative, suggesting negative local and spatial spillovers of public health concern on average; third, in column (8), the short-run local spillover (SR_Direct_lnphcb2) and short-run spatial spillover (SR_Indirect_lnphcb2) of squared public health concern are both negative, suggesting inverse U-shaped local and spatial spillovers of public health concern in general. Thus, in the short run, both low-risk incident and high-risk incident have negative local and spatial spillovers to high-risk food price risk; on average, public health concern over food safety has negative local and spatial spillovers to high-risk food price risk; in general, public health concern over food safety has inverse U-shaped local and spatial spillovers to high-risk food price risk.

(ii) In columns (7)–(8), the long-run local spillovers (LR_Direct_lrid, LR_Direct_hrid, LR_Direct_lnphcb, and LR_Direct_lnphcb2) and long-run spatial spillovers (LR_Indirect_lrid, LR_Indirect_hrid, LR_Indirect_lnphcb, and LR_Indirect_lnphcb2) are all statistically insignificant, suggesting no significant local or spatial spillover of food safety incident and public health concern. Thus, in the long run, neither low-risk incident nor high-risk incident has a significant local or spatial spillover to high-risk food price risk; public health concern over food safety has no significant local or spatial spillover to high-risk food price risk.

Fourth, turning point for public health concern: We compute the turning point for public health concern in the inverse U-shape using the main coefficients (β_3_ and β_4_) following Wooldridge [35]. We obtain the turning point values for public health concern (turning_lnphcb) from column (4) (dynamic SAR for low-risk food) and column (8) (dynamic SAR for high-risk food), which are −2.3420 and −2.9651 (well within the range of the observed data on lnphcb), respectively, as also reported in Table 4.

**Figure 4:**
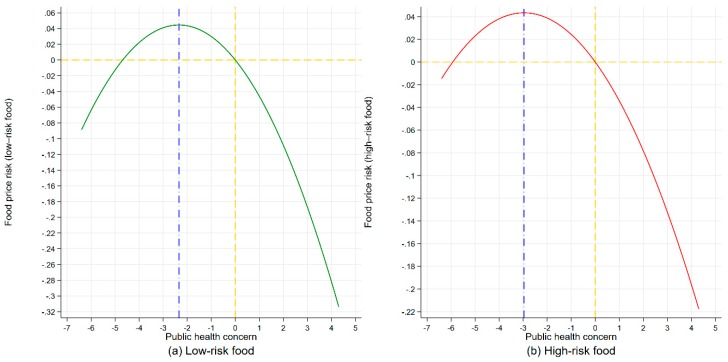
(**a**) Plot of marginal effect of public health concern on low-risk food price risk from column (4) of Table 4; and (**b**) Plot of marginal effect of public health concern on lnhrfp from column (8) of Table 4. Dot-dashed blue line denotes lnphcb turning point reference line. Dashed gold line denotes 0 reference line. *Source:* Authors’ original calculations using Stata.

Fifth, plots of marginal effects of public health concern: We further plot the marginal effects of public health concern on low-risk food price risk (column (4) of Table 4) and high-risk food price risk (column (8) of Table 4), respectively, using the main coefficients (β_3_ and β_4_) and turning point values for public health concern (turning_lnphcb), as depicted in Figure 4. In both panels, the curves exhibit a pronounced inverse U-shaped pattern, lending further support to hypothesis H2.

In summary, these results show that (i) food safety incident alone only triggers high-risk food price risk, not low-risk food price risk; and (ii) public health concern amplifies nonlinear food price risk triggered by food safety incident. Overall, food safety incident itself does not necessarily determine food price risk, whereas it is actually public health concern that directly causes nonlinear food price risk. Therefore, hypotheses H1–H2 are generally supported.

### 4.3. Further Analysis of Heterogeneous Food Price Risk Mechanism: Hypotheses H3–H4

#### 4.3.1. Further Analysis of Heterogeneous Food Price Risk Moderation: Hypothesis H3

By allowing for the interaction between food safety incident and public health concern following Hayes [36], we seek to test whether expected moderation exists, that is, whether food safety incident negatively moderates the negative local and spatial spillovers of public health concern over food safety to food price risk, which is heterogeneous in low- and high-risk food and incidents (hypothesis H3). We estimate Equations (5) and (6) (dynamic SAR, the first-best fit model) using QML estimator, excluding and including the interaction terms between low risk incident and public health concern (lrid*lnphcb), and high risk incident and public health concern (hrid*lnphcb), respectively, all with FE, as reported in Table 5: (i) columns (1)–(2) report estimates of Equation (5) only including lrid and lnphcb (Step 1), and Equation (6) further including lrid*lnphcb (Step 2), respectively, with lnlrfp as DV; (ii) columns (3)–(4) report estimates of Equation (5) only including hrid and lnphcb (Step 1), and Equation (6) further including hrid*lnphcb (Step 2), respectively, with lnlrfp as DV; and (iii) columns (5)–(8) report estimates with lnhrfp as DV.

First, spatial specification diagnostic tests: Using spatial coefficients (ρ), we find that all columns are statistically significant, suggesting that spatially lagged effects of food price risk should be accounted for.

Second, empirical analysis for low-risk food: (columns (2) and (4)). (i) In column (2), the short-run local spillover (SR_Direct_lrid*lnphcb), short-run spatial spillover (SR_Indirect_lrid*lnphcb), long-run local spillover (LR_Direct_lrid*lnphcb), and long-run spatial spillover (LR_Indirect_lrid*lnphcb) of interaction lrid × lnphcb are all statistically insignificant, suggesting no significant moderation of local or spatial spillover of public health concern by low-risk incident. Thus, whether in the short run or in the long run, low-risk incident doesn’t moderate the local or spatial spillover of public health concern over food safety to low-risk food price risk.

(ii) In column (4), the short-run local spillover (SR_Direct_hrid*lnphcb) and short-run spatial spillover (SR_Indirect_hrid*lnphcb) of interaction hrid × lnphcb are both negative, suggesting negative moderation of local and spatial spillovers of public health concern by high-risk incident. Thus, in the short run, high-risk incident negatively moderates the negative local and spatial spillovers of public health concern over food safety to low-risk food price risk.

(iii) In column (4), the long-run local spillover (LR_Direct_hrid*lnphcb) of interaction hrid × lnphcb is negative, and long-run spatial spillover (LR_Indirect_hrid*lnphcb) of interaction hrid × lnphcb is statistically insignificant, suggesting negative moderation of local spillover but no significant moderation of spatial spillover of public health concern by high-risk incident. Thus, in the long run, high-risk incident negatively moderates the negative local spillover of public health concern over food safety to low-risk food price risk; high-risk incident doesn’t moderate the spatial spillover of public health concern over food safety to low-risk food price risk.

Third, empirical analysis for high-risk food: (columns (6) and (8)). (i) In column (6), the short-run local spillover (SR_Direct_lrid*lnphcb), short-run spatial spillover (SR_Indirect_lrid*lnphcb), long-run local spillover (LR_Direct_lrid*lnphcb), and long-run spatial spillover (LR_Indirect_lrid*lnphcb) of interaction lrid × lnphcb are all statistically insignificant, suggesting no significant moderation of local or spatial spillover of public health concern by low-risk incident. Thus, whether in the short run or in the long run, low-risk incident doesn’t moderate the local or spatial spillover of public health concern over food safety to high-risk food price risk.

(ii) In column (8), the short-run local spillover (SR_Direct_hrid*lnphcb) and short-run spatial spillover (SR_Indirect_hrid*lnphcb) of interaction hrid × lnphcb are both negative, suggesting negative moderation of local and spatial spillovers of public health concern by high-risk incident. Thus, in the short run, high-risk incident negatively moderates the negative local and spatial spillovers of public health concern over food safety to high-risk food price risk.

(iii) In column (8), the long-run local spillover (LR_Direct_hrid*lnphcb) and long-run spatial spillover (LR_Indirect_hrid*lnphcb) are both statistically insignificant, suggesting no significant moderation of local or spatial spillover of public health concern by high-risk incident. Thus, in the long run, high-risk incident doesn’t moderate the local or spatial spillover of public health concern over food safety to high-risk food price risk.

In summary, these results show that high-risk incident intensifies negative pressure of public health concern on food price risk. Therefore, hypothesis H3 is generally supported.

#### 4.3.2. Further Analysis of Heterogeneous Food Price Risk Mediation: Hypothesis H4

By using an extended spatial causal steps approach motivated by the Baron and Kenny method [33], we seek to test whether expected mediation exists; that is, whether public health concern over food safety mediates the negative local and spatial spillovers of food safety incident to food price risk, which is heterogeneous in low- and high-risk food and incidents (hypothesis H4). We estimate Equations (7)–(9) (dynamic SAR, the first-best fit model) using QML estimator, following the causal steps approach in Baron and Kenny [33], respectively, all with FE, as reported in Table 6: (i) columns (1)–(3) report estimates of Equation (7) including lrid with lnlrfp as DV (Step 1), Equation (8) including lrid with lnphcb as DV (Step 2), and Equation (9) including lrid and lnphcb with lnlrfp as DV (Step 3); (ii) columns (4)–(6) report estimates of Equation (7) including hrid with lnlrfp as DV (Step 1), Equation (8) including hrid with lnphcb as DV (Step 2), and Equation (9) including hrid and lnphcb with lnlrfp as DV (Step 3); and (iii) columns (7)–(12) report estimates with lnhrfp as DV.

First, spatial specification diagnostic tests: Using spatial coefficients (ρ), we find that all columns are statistically significant, suggesting that spatially lagged effects of food price risk should be accounted for.

Second, empirical analysis for low-risk food: (columns (1)–(6)). In columns (1) and (4), the short-run local spillovers (SR_Direct_lrid and SR_Direct_hrid), short-run spatial spillovers (SR_Indirect_lrid and SR_Indirect_hrid), long-run local spillovers (LR_Direct_lrid and LR_Direct_hrid), and long-run spatial spillovers (LR_Indirect_lrid and LR_Indirect_hrid) of lrid and hrid with lnlrfp as DV are all statistically insignificant (thereby not passing Step 1), suggesting no significant mediation of local or spatial spillover of low-risk incident and high-risk incident by public health concern. Thus, whether in the short run or in the long run, public health concern over food safety doesn’t mediate the local or spatial spillover of low-risk incident to low-risk food price risk; public health concern over food safety doesn’t mediate the local or spatial spillover of high-risk incident to low-risk food price risk.

Third, empirical analysis for high-risk food: (columns (7)–(12)). (i) First, in columns (7) and (10), the short-run local spillovers (SR_Direct_lrid and SR_Direct_hrid) and short-run spatial spillovers (SR_Indirect_lrid and SR_Indirect_hrid) of lrid and hrid with lnhrfp as DV are all negative (thereby passing Step 1); second, in columns (8) and (11), the short-run local spillovers (SR_Direct_lrid and SR_Direct_hrid) and short-run spatial spillovers (SR_Indirect_lrid and SR_Indirect_hrid) of lrid and hrid with lnphcb as DV are all negative (thereby passing Step 2); third, in columns (9) and (12), the short-run local spillovers (SR_Direct_lnphcb) and short-run spatial spillovers (SR_Indirect_lnphcb) of lnphcb with lnhrfp as DV are both negative (thereby passing Step 3); fourth, in columns (9) and (12), SR_Direct_lrid, SR_Direct_hrid, and SR_Indirect_hrid are all negative, while SR_Indirect_lrid is statistically insignificant, suggesting partial mediation of local spillover of low-risk incident by public health concern, complete mediation of spatial spillover of low-risk incident by public health concern, and partial mediation of local and spatial spillovers of high-risk incident by public health concern. Thus, in the short run, public health concern over food safety partially mediates the negative local spillover and completely mediates the negative spatial spillover of low-risk incident to high-risk food price risk; public health concern over food safety partially mediates the negative local and spatial spillovers of high-risk incident to high-risk food price risk.

(ii) In columns (7) and (10), the long-run local spillovers (LR_Direct_lrid and LR_Direct_hrid) and long-run spatial spillovers (LR_Indirect_lrid and LR_Indirect_hrid) of lrid and hrid with lnhrfp as DV are all statistically insignificant (thereby not passing Step 1), suggesting no significant mediation of local or spatial spillover of low-risk incident and high-risk incident by public health concern. Thus, in the long run, public health concern over food safety doesn’t mediate the local or spatial spillover of low-risk incident to high-risk food price risk; public health concern over food safety doesn’t mediate the local or spatial spillover of high-risk incident to high-risk food price risk.

Fourth, mediation effect and ratio of mediation effect to total effect: We further compute mediation effect (ME) and ratio of mediation effect to total effect (MR) using the main coefficients following Mackinnon, Warsi, and Dwyer [37]. We obtain ME and MR from columns (9) and (12) where mediation is statistically significant, as also reported in Table 6. Results show that, (i) for column (9), the mediation effect of spillover of low-risk incident to high-risk food price risk by public health concern is −0.0162 (ME = −0.0162), accounting for 12.45% of the total spillover (MR = 0.1245); and (ii) for column (12), the mediation effect of spillover of high-risk incident to high-risk food price risk by public health concern is −0.0075 (ME = −0.0075), accounting for 6.44% of the total spillover (MR = 0.0644).

In summary, these results show that food safety incident indirectly affects high-risk food price risk through public health concern. Therefore, hypothesis H4 is generally supported.

Moreover, we subject our analyses to a variety of robustness checks as described in Table 2, finding that all results are robust to using alternative measurement of food safety incident, alternative measurement of public health concern, and alternative spatial weighting matrix.

## 5. Discussion

We discuss the relation with the existing literature. Throughout, we are more concerned about short-run spillovers than long-run spillovers because, consistent with our theoretical framework, food safety risk, as a temporary exogenous shock, may cause rapid food demand deficiency and sharp food price pressure in the short run, but can result in slow food demand recovery and gradual food price reversal in the long run, which is likely to be insignificant.

### 5.1. Discussion on Heterogeneous Food Price Risk Nonlinearity: Hypotheses H1–H2

**Figure 5:**
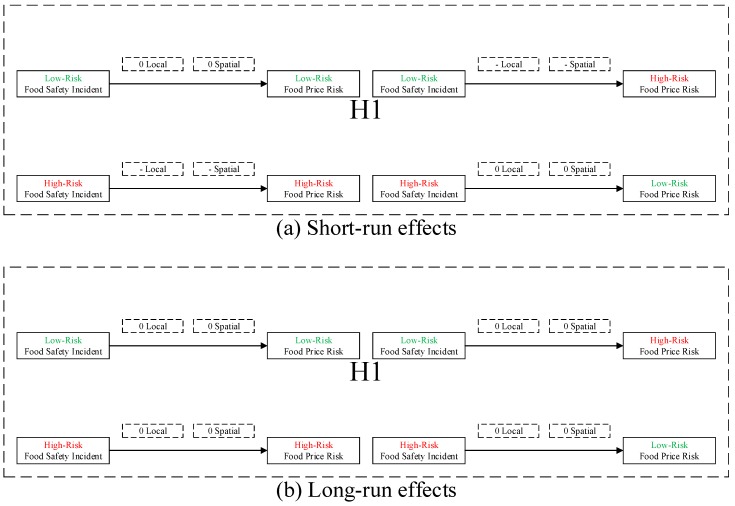
Test results for hypothesis H1. The symbols “0” and “-” show “insignificant” and “negative” spillovers, respectively. *Source:* Originally developed by the authors.

First, the test results in Table 4 are generally consistent with hypothesis H1 that food safety incident has negative local and spatial spillovers to high-risk food price risk, which is heterogeneous in low- and high-risk food. More specifically: (i) In the short run, for low-risk food, neither low-risk incident nor high-risk incident has a significant local or spatial spillover to food price risk; for high-risk food, both low-risk incident and high-risk incident have negative local and spatial spillovers to food price risk. (ii) In the long run, neither low-risk incident nor high-risk incident has a significant local or spatial spillover to food price risk. We illustrate test results for hypothesis H1 in Figure 5.

In the wake of food safety incidents [38], potentially followed by food safety scares [39], related food price may fluctuate more drastically, and transmit throughout the supply chain and across areas, which can lead to local and spatial food price risk [40], where market prices of heterogeneous food may respond differently to the same realizations of the shocks. Specifically, (i) for low-risk food, food safety incidents might not heighten consumers’ food risk perception [41] due to relatively low infectivity, thus not influencing related food price risk; whereas (ii) for high-risk food, consumers might fear being infected and reduce food consumption, thereby triggering related food price risk.

**Figure 6:**
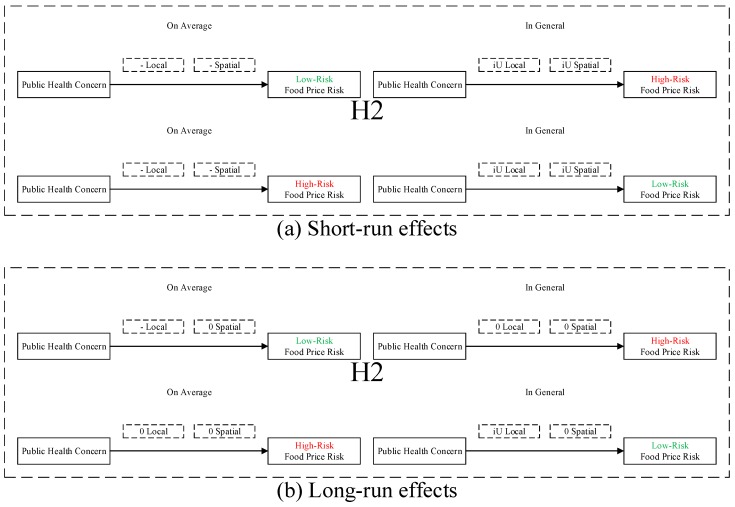
Test results for hypothesis H2. The symbols “0”, “-”, and “iU” show “insignificant”, “negative”, and “inverse U-shaped” spillovers, respectively. *Source:* Originally developed by the authors.

Second, the test results in Table 4 are generally consistent with hypothesis H2 that public health concern over food safety has nonlinear local and spatial spillovers to food price risk, which is heterogeneous in low- and high-risk food. More specifically: (i) In the short run, public health concern over food safety has negative local and spatial spillovers to food price risk on average, and inverse U-shaped local and spatial spillovers to food price risk in general. (ii) In the long run, public health concern over food safety has no significant spatial spillover to food price risk; for low-risk food, public health concern over food safety has a negative local spillover to food price risk on average, and an inverse U-shaped local spillover to food price risk in general; for high-risk food, public health concern over food safety has no significant local spillover to food price risk. We illustrate test results for hypothesis H2 in Figure 6.

After being officially released, source information [42] on food safety risk may be extensively covered by online media (Weibo, WeChat, Facebook, Twitter, etc.) [43] who act as online opinion leaders [44], arousing widespread public health concern over food safety [45], which amplifies the impact of food safety risk by drawing consumer attention to the incident [46], imposing price pressure [47] on food price. Specifically, (i) when the magnitude of public health concern is relatively low, consumers may only have limited attention to food safety risk [48], and the decrease in producers’ food supply can be larger than the decrease in consumers’ food demand, resulting in an increase in local and neighboring food price which marginally decreases; and (ii) when the magnitude of public health concern is relatively high, consumers may have excessive attention to food safety incident [49], and the decrease in consumers’ food demand may far exceed the decrease in producers’ food supply, resulting in an decrease in local and neighboring food price which marginally increase.

Third, we discuss the relationship between our results and aspects of this literature.

(i) Our finding that food safety incident itself does not necessarily determine food price risk, is roughly associated with Han and Xu [50] claiming that avian influenza epidemic has no significant impact on broiler price risk; and associated with Yi et al. [5] suggesting that neither poultry infection nor human infection with avian influenza outbreak has a significant spillover to broiler price risk.

However, previous literature typically conducts empirical analyses in settings with homogeneous risk levels of food products and food safety incidents, and potential heterogeneity in causal effects with respect to different risk levels of food products and food safety incidents could not be fully captured. Whereas we explicitly differentiate between low- and high-risk food and incidents, and further assess food price risk spillover in a setting with heterogeneous food safety risk levels, arguing that food safety incident alone only directly causes high-risk food price risk, not low-risk food price risk. Therefore, our finding complements our knowledge on the diverse impacts of food safety incident on heterogeneous food price risk, suggesting that food risk level matters for the food price risk spillover of exogenous food safety shocks to consumer markets.

(ii) Our finding that public health concern over food safety spills over to food price risk, is roughly consistent with a large body of literature claiming that food safety risk can cause significant food price risk in consumer markets [11].

However, previous literature typically measures food safety risk simply as a whole without differentiating its potential components. Whereas we decompose food safety risk into food safety incident (objective incident component) and public health concern over food safety (subjective concern component), highlighting that it is public health concern that amplifies the impact of food safety incident on related food price risk. Therefore, our finding elucidates heterogeneous factors of food safety risk influencing related food price, which are obscured in previous literature.

(iii) Our finding that food price risk is significantly responsive to exogenous food safety shocks, also roughly accords with previous research indicating that facing a large external shock, food price fluctuates greatly and frequently [51], and that food price risk is vertically transmitted across markets [52].

However, previous research typically uses time-series data based on a single district to analyze food price volatility and food price vertical transmission; whereas, we adopt a long panel data set consisting of 30 provinces from China covering November 2007 to November 2017 to identify the causal effects of food safety incident and public health concern on food price risk. Therefore, our finding can address potential omitted variable concerns from which time-series techniques may suffer.

(iv) Our finding that public health concern over food safety exerts a substantial spatial impact on consumer markets, again, roughly supports prior work suggesting that there exist food spatial market integration and food spatial price dispersion across regional markets [53].

However, prior work typically focuses on testing if the law of one price (LOP) holds spatially, and seldom explores food price risk spatial spillover effects; even though some work investigates the spatial spillovers between food crops prices and yields using annual panel data [54], the evidence for food price risk of animal products at the monthly level or higher levels is particularly scarce. Whereas we use exogenous avian influenza shocks to China’s poultry markets with monthly spatial panel data to quantify the local and spatial spillovers of food safety incident and public health concern to food price risk. Therefore, our finding deepens our understanding of how food safety risk spills over to local and neighboring consumer markets.

### 5.2. Discussion on Heterogeneous Food Price Risk Mechanism: Theoretical H3–H4

#### 5.2.1. Discussion on Heterogeneous Food Price Risk Moderation: Hypothesis H3

**Figure 7:**
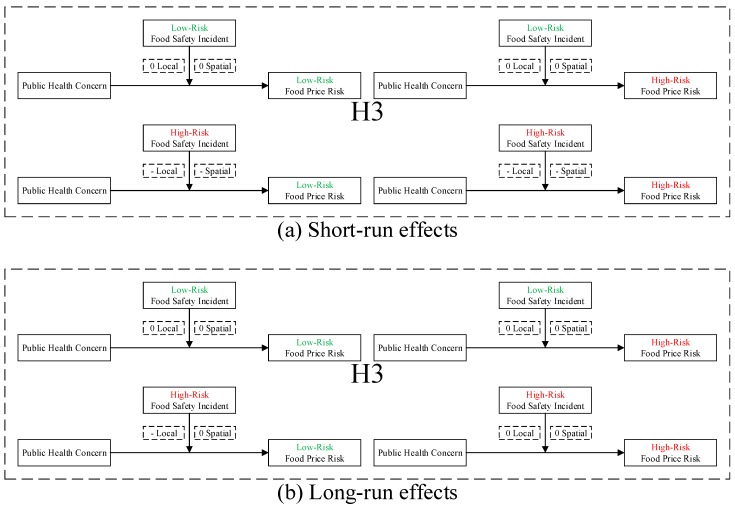
Test results for hypothesis H3. The symbols “0” and “-” show “insignificant” and “negative” moderation, respectively. *Source:* Originally developed by the authors.

First, the test results in Table 5 are generally consistent with hypothesis H3 that food safety incident negatively moderates the negative local and spatial spillovers of public health concern over food safety to food price risk, which is heterogeneous in low- and high-risk food and incidents. More specifically: (i) In the short run, low-risk incident doesn’t moderate the local or spatial spillover of public health concern over food safety to food price risk; high-risk incident negatively moderates the negative local and spatial spillovers of public health concern over food safety to food price risk. (ii) In the long run, low-risk incident doesn’t moderate the local or spatial spillover of public health concern over food safety to food price risk; high-risk incident doesn’t moderate the spatial spillover of public health concern over food safety to food price risk; for low-risk food, high-risk incident negatively moderates the negative local spillover of public health concern over food safety to food price risk; for high-risk food, high-risk incident doesn’t moderate the local spillover of public health concern over food safety to food price risk. We illustrate test results for hypothesis H3 in Figure 7.

Heterogeneous food safety incidents may influence the impact of public health concern over food safety on related food price risk, even when facing the same magnitude of public health concern, in that under the same extent of online media coverage [55], high-risk incident may induce higher risk perception than do low-risk incident, leading to larger influence on the effect of public health concern [56]. Specifically, (i) when facing low-risk incidents, consumers might not care about the incidents because they are relatively less dangerous or infectious, thus not moderating the effects of public health concern on related food price risk; and (ii) when facing high-risk incidents, consumers may have higher food risk perception and greatly reduce food consumption, thereby negatively moderating the effects of public health concern on related food price risk.

Second, we discuss the relationship between our results and aspects of this literature.

Our finding that there exist moderation effects in the spillover of food safety risk to consumer markets, is roughly consistent with Zhang et al. [57] claiming that media and gender moderate the impacts of risk governance (RG) and risk perception (RP) on personal risk prevention behavior (PRP) with regard to food safety issues; and roughly consistent with Prentice, Chen, and Wang [58] suggesting that consumers’ purchase styles moderate the impact of food product quality assessment on food purchase intention.

However, previous literature typically explores moderation effects using food consumer decision models without allowing for food price risk. Whereas we account for food price risk moderation by including the interaction terms between public health concern and low- and high-risk incidents, showing that high-risk incident negatively moderates negative local and spatial spillovers of public health concern to food price risk. Therefore, our finding deepens our understanding of food price risk moderation facing food scares.

#### 5.2.2. Discussion on Heterogeneous Food Price Risk Mediation: Hypothesis H4

**Figure 8:**
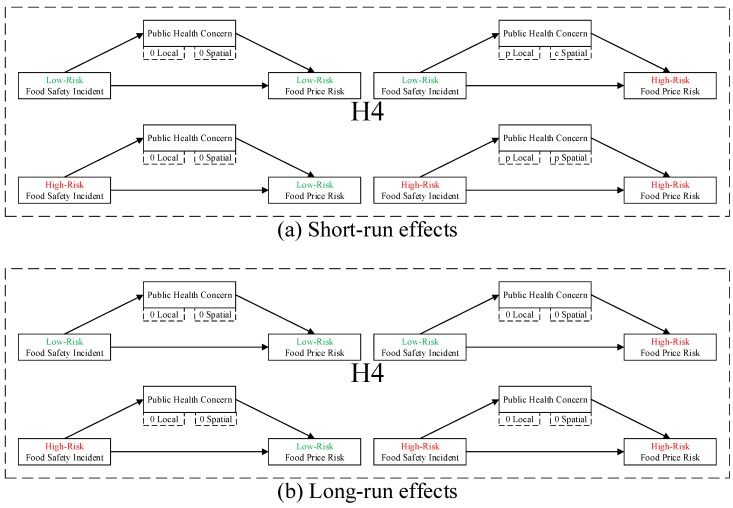
Test results for hypothesis H4. The symbols “0”, “p”, and “c” show “insignificant”, “partial”, and “complete” mediation, respectively. *Source:* Originally developed by the authors.

First, the test results in Table 6 are generally consistent with hypothesis H4 that public health concern over food safety mediates the negative local and spatial spillovers of food safety incident to food price risk, which is heterogeneous in low- and high-risk food and incidents. More specifically: (i) In the short run, for low-risk food, public health concern over food safety doesn’t mediate the local or spatial spillover of food safety incident to food price risk; for high-risk food, public health concern over food safety partially mediates the negative local spillover and completely mediates the negative spatial spillover of low-risk incident to food price risk, and partially mediates the negative local and spatial spillovers of high-risk incident to food price risk. (ii) In the long run, public health concern over food safety doesn’t mediate the local or spatial spillover of food safety incident to food price risk. (iii) The mediation of low-risk incident’s spillover to high-risk food price risk by public health concern accounts for 12.45% of the total spillover, while the mediation of high-risk incident’s spillover to high-risk food price risk by public health concern accounts for 6.44% of the total spillover. We illustrate test results for hypothesis H4 in Figure 8.

Accounting for limited attention [26] and two-step flow of communication [27], food safety incident may affect related food price risk through public health concern, in that: (i) source information on food safety incident, which is initially released by the authorities during first-step flow, may only receive limited consumers’ attention before being extensively covered by online media; (ii) after being covered and disseminated by online media, source information on food safety incident gets amplified by public health concern over food safety during second-step flow, leading to excessive consumers’ attention; and (iii) clustered public health concern can give rise to local and spatial food price pressure. Moreover, the causal path may be heterogeneous in terms of diverse food and incidents. Specifically, (i) for low-risk food, as food safety incidents of this kind generally do not draw consumers’ attention [59] and affect consumers’ behavior [60], public health concern over food safety does not mediate the effect of food safety incident on related food price risk; whereas (ii) for high-risk food, since consumers can respond to this kind of food safety incidents, which may lead to overreaction in food markets, public health concern over food safety mediates the effect of food safety incident on related food price risk.

Second, we discuss the relationship between our results and aspects of this literature.

Our finding that there exist mediation effects in the spillover of food safety risk to consumer markets, is roughly consistent with Yu, Sirsat, and Neal [61] suggesting that job satisfaction and self-efficacy completely mediate the impact of food safety training on food safety whistle-blowing; and roughly consistent with Sanlier and Baser [62] maintaining that food safety attitude mediates the impact of food safety knowledge on food safety behavior.

However, previous literature typically investigates mediation effects via food consumer decision models regardless of food price risk. Whereas we further consider food price mediation by implementing the causal steps approach following Baron and Kenny [33], claiming that public health concern partially mediates negative local and completely mediates negative spatial spillovers of low-risk incident to high-risk food price risk, and partially mediates negative local and spatial spillovers of high-risk incident to high-risk food price risk. Therefore, our finding provides a deeper understanding of food price risk mediation in the wake of a food scare.

## 6. Conclusions

By documenting several key stylized facts, our article complements and extends the literature on the impact of food safety risk on consumer markets in public health economics.

Overall, food safety incident itself does not necessarily determine food price risk, whereas it is actually public health concern that directly causes nonlinear food price risk.

First, food safety incident alone only triggers high-risk food price risk, not low-risk food price risk. (i) For low-risk food, neither low-risk incident nor high-risk incident has a significant local or spatial spillover to food price risk; and (ii) for high-risk food, both low-risk incident and high-risk incident have negative local and spatial spillovers to food price risk.

Second, public health concern amplifies nonlinear food price risk triggered by food safety incident. Public health concern over food safety has negative local and spatial spillovers to food price risk on average, and inverse U-shaped local and spatial spillovers to food price risk in general.

Third, high-risk incident intensifies negative pressure of public health concern on food price risk. (i) Low-risk incident doesn’t moderate the local or spatial spillover of public health concern over food safety to food price risk; and (ii) high-risk incident negatively moderates the negative local and spatial spillovers of public health concern over food safety to food price risk.

Fourth, food safety incident indirectly affects high-risk food price risk through public health concern. (i) For low-risk food, public health concern over food safety doesn’t mediate the local or spatial spillover of food safety incident to food price risk; (ii) for high-risk food, public health concern over food safety partially mediates the negative local spillover and completely mediates the negative spatial spillover of low-risk incident to food price risk, and partially mediates the negative local and spatial spillovers of high-risk incident to food price risk; and (iii) the mediation of low-risk incident’s spillover to high-risk food price risk by public health concern accounts for 12.45% of the total spillover, while the mediation of high-risk incident’s spillover to high-risk food price risk by public health concern accounts for 6.44% of the total spillover.

Our findings have significant policy implications for food markets in global perspectives in public health economics. (i) On price risk nonlinearity: As public health concern over food safety exerts an food-specific inverse U-shaped price pressure on related food price, causing nonlinear food price risk, in the wake of a food scare, regulatory authorities should closely monitor the magnitude of public health concern, especially when it is approaching or exceeding the turning point value for public health concern, since food price risk shifts from a decreasing positive marginal effect on the left-hand side to an increasing negative marginal effect on the right-hand side, which aggravates food price risk. (ii) On price risk moderation: As outbreak of high-risk rather than low-risk incident deteriorates price pressure of public health concern on food price risk, regulatory authorities should focus more on high-risk incident while addressing food price risk issues facing food scares. (iii) On price risk mediation: As food safety incident indirectly affects high-risk rather than low-risk food price risk through public health concern, regulatory authorities should pay more attention to the price risk of high-risk food facing a food scare.

Our analysis points to several directions for future research in public health economics. (i) On theoretical framework: We develop our theoretical framework of causality based on the theories of limited attention and two-step flow of communication to capture the causal effects of food safety incident and public health concern on food price risk at the macro level. Future work could extend our theoretical framework by allowing for heterogeneity in consumer attention and behavior at the micro level. (ii) On spatial nonlinearity: We include the squared term of public health concern to account for the nonlinear spatial relationship between food price risk and public health concern over food safety, showing an inverse U-shape. Future work could extend our quadratic function to panel threshold (PT) models or unconditional quantile regression models (UQR) to further investigate the potential nonlinearity in the relationship. (iii) On spatial heterogeneity: We assess spatial heterogeneity in food price risk spillover by differentiating between low- and high-risk food and incidents, using static and dynamic spatial panel-data models such as SDM, dynamic SDM, SAR, dynamic SAR, SAC, and SEM. Future work could allow for potential spatiotemporal heterogeneity in each area by using geographically and temporally weighted regression (GTWR) models.

## Figures and Tables

**Table 1:** Introduction summary.

Subsection	Summary
Research Background	Food safety incident arouses public health concern, causing food price risk
Research Motivations	To understand how food safety risk affects food price risk
Research Questions	(i) Causal effects with information communication; (ii) heterogeneous food price risk spillover
Research Methods	(i) Variable decomposition and theoretical framework; (ii) avian influenza shocks as natural experiments
Research Significance	Filling the knowledge gaps on theory and evidence
Defining Terms	Defining certain key terms
Research Objectives	Stating the purpose of research
Article Structure	Providing an overview of the article structure

*Source:* Originally developed by the authors.

**Table 2:** Summary of variable measurements and data sources.

Category	Concept	Variable Name	Measurement	Indicator	Source
Dependent variable	Food price risk	lnlrfp	Low–risk food price risk	Log dressed broiler price	CAAA
(Food price risk spillover)		lnhrfp	High–risk food price risk	Log live broiler price	CAAA
Key independent variable	Food safety incident	lrid	Low–risk food safety incident	Poultry infection with avian influenza incident dummy	Official Veterinary Bulletin
(Food safety risk)		hrid	High–risk food safety incident	Human infection with avian influenza incident dummy	Disease Surveillance
		lnlric	Low–risk food safety case (robustness)	Log poultry infection with avian influenza case	Official Veterinary Bulletin
		lnhric	High–risk food safety case (robustness)	Log human infection with avian influenza case	Disease Surveillance
	Public health concern	lnphcb	Public health concern by Baidu	Log Baidu search volume on avian influenza	Baidu Search
		lnphcb2	Squared public health concern by Baidu	Squared log Baidu search volume on avian influenza	Baidu Search
		lnphcg	Public health concern by Google (robustness)	Log Google search volume on avian influenza	Google Search
		lnphcg2	Squared public health concern by Google (robustness)	Squared log Google search volume on avian influenza	Google Search
Price control variable		lnfp	Feed price	Log broiler feed price	CAAA
(Industrial vertical price transmission)		lncp	Chick price	Log broiler chick price	CAAA
		lnhrfp	Live broiler price (robustness)	Log live broiler price	CAAA
		lnlrfp	Dressed broiler price (robustness)	Log dressed broiler price	CAAA
		lnpp	Pork price	Log pork price	CAAA
Supply and demand control variable		lno	Aggregate poultry output	Log aggregate poultry output	EPS China Data
(Market supply and demand)		lnu	Urban poultry consumption	Log urban poultry consumption	EPS China Data
		lnr	Rural poultry consumption	Log rural poultry consumption	EPS China Data
Spatial weighting matrix		W^s^	Squared idistance matrix	Squared inverse-distance spatial weighting matrix	GADM data
(Interregional horizontal price transmission)		W^e^	Exponential idistance matrix (robustness)	Exponential inverse-distance spatial weighting matrix	GADM data

*Source:* All data are originally collected by the authors.

**Table 3:** Summary statistics.

VarName	Obs	Mean	SD	Min	P25	Median	P75	Max	Skewness	Kurtosis
lnlrfp	3630	0.0000	0.9960	−5.0843	−0.7234	0.0618	0.7480	3.4881	−0.2304	3.0000
lnhrfp	3630	0.0000	0.9960	−4.9214	−0.6609	0.1223	0.7346	5.0134	−0.4542	3.9352
lrid	3630	0.0146	0.1200	0.0000	0.0000	0.0000	0.0000	1.0000	8.0935	66.5054
hrid	3630	0.0752	0.2638	0.0000	0.0000	0.0000	0.0000	1.0000	3.2215	11.3780
lnlric	3630	−11.2445	2.2159	−11.5129	−11.5129	−11.5129	−11.5129	7.6728	8.1912	68.4502
lnhric	3630	−10.5739	3.3019	−11.5129	−11.5129	−11.5129	−11.5129	2.3026	3.2515	11.6488
lnphcb	3630	0.0000	0.9960	−6.3867	−0.3994	−0.0225	0.3263	4.3000	−0.3670	9.5866
lnphcb2	3630	0.9917	2.9065	0.0000	0.0359	0.1282	0.3453	40.7897	5.8380	51.1054
lnphcg	3630	0.0000	0.9960	−2.4759	−0.7836	−0.1782	0.7888	3.0215	0.2770	2.3000
lnphcg2	3630	0.9917	1.1309	0.0000	0.1977	0.6172	1.3560	9.1292	2.0964	8.8429
lnfp	3630	0.0000	0.9960	−2.9780	−0.6490	0.1504	0.7813	3.5090	−0.4305	2.4208
lncp	3630	0.0000	0.9960	−3.2307	−0.7185	0.0805	0.7172	3.3564	−0.1455	2.6085
lnpp	3630	0.0000	0.9960	−2.6673	−0.6637	0.0067	0.7602	2.3435	−0.2028	2.6035
lno	3630	0.0000	0.9960	−3.8941	−0.8146	0.2220	0.8382	3.4258	−0.4913	2.4515
lnu	3630	0.0000	0.9960	−4.9970	−0.8544	0.2819	0.7217	2.7168	−0.9854	4.3730
lnr	3630	0.0000	0.9960	−3.2582	−0.8385	−0.0496	0.8958	1.8784	0.0948	1.8955

*Notes:* Summary statistics for processed data. *Source:* Authors’ original calculations using Stata.

**Table 4:** Benchmark analysis of heterogeneous food price risk nonlinearity.

Variable	(1)	(2)	(3)	(4)	(5)	(6)	(7)	(8)
	L_dynSDM_1	L_dynSDM_2	L_dynSAR_1	L_dynSAR_2	H_dynSDM_1	H_dynSDM_2	H_dynSAR_1	H_dynSAR_2
lrid (β_1_)	0.0093	0.0108	0.0108	0.0177	−0.1110 *	−0.1146 *	−0.1088 *	−0.1045 *
hrid (β_2_)	−0.0228	−0.0194	−0.0334	−0.0254	−0.0951 **	−0.0948 **	−0.1044 **	−0.0999 **
lnphcb (β_3_)	−0.0236 ***	−0.0259 ***	−0.0337 ***	−0.0380 ***	−0.0177 *	−0.0156	−0.0266 ***	−0.0293 ***
lnphcb2 (β_4_)		−0.0057**		−0.0081 ***		−0.0018		−0.0049 **
Wy (ρ)	0.4913 ***	0.4809 ***	0.5019 ***	0.4931 ***	0.4693 ***	0.4583 ***	0.4790 ***	0.4739 ***
SR_Direct_lrid	−0.0069	−0.0034	0.0114	0.0197	−0.1220 *	−0.1226 **	−0.1158 *	−0.1104 *
SR_Direct_hrid	−0.0240	−0.0177	−0.0350	−0.0253	−0.1026 **	−0.0983 **	−0.1111 **	−0.1044 **
SR_Direct_lnphcb	−0.0279 ***	−0.0296 ***	−0.0349 ***	−0.0393 ***	−0.0205 **	−0.0180	−0.0270 ***	−0.0297 ***
SR_Direct_lnphcb2		−0.0069 ***		−0.0085 ***		−0.0030		−0.0050 **
SR_Indirect_lrid	−0.2843	−0.2694	0.0116	0.0181	−0.1672	−0.1313	−0.0986 *	−0.0929
SR_Indirect_hrid	−0.0323	0.0028	−0.0314	−0.0215	−0.1217	−0.0822	−0.0974 *	−0.0898 *
SR_Indirect_lnphcb	−0.0868 ***	−0.0801 ***	−0.0321 ***	−0.0347 ***	−0.0706 ***	−0.0696 ***	−0.0227 ***	−0.0243 ***
SR_Indirect_lnphcb2		−0.0223 ***		−0.0076 ***		−0.0246 ***		−0.0040 **
LR_Direct_lrid	0.0361	0.0541	0.0577	0.0989	−2.0319	−1.2032	−1.8116	−1.5079
LR_Direct_hrid	−0.1200	−0.0948	−0.1776	−0.1273	−1.5385	−1.0353	−1.7073	−1.3615
LR_Direct_lnphcb	−0.1269 ***	−0.1365 ***	−0.1768 ***	−0.1978 ***	−0.1787	−0.1394	−0.4184	−0.3589
LR_Direct_lnphcb2		−0.0307 **		−0.0430 ***		0.0024		−0.0661
LR_Indirect_lrid	−0.9297	−0.8769	0.0230	0.0228	0.7158	0.0348	0.8705	0.6167
LR_Indirect_hrid	−0.0540	0.0539	−0.0237	−0.0101	0.4889	0.2091	0.7307	0.4989
LR_Indirect_lnphcb	−0.2346 *	−0.2030 *	−0.0316	−0.0302	−0.2137	−0.2340	0.2098	0.1497
LR_Indirect_lnphcb2		−0.0590 **		−0.0067		−0.1204		0.0306
BIC	3260.1037	3290.8175	3245.2175	3270.1863	3138.4065	3171.9346	3119.6369	3154.9565
turning_lnphcb				−2.3420				−2.9651

*Notes:* All control variables are included but not reported. * *p* < 0.10, ** *p* < 0.05, *** *p* < 0.01. *Source:* Authors’ original calculations using Stata.

**Table 5:** Further Analysis of heterogeneous food price risk moderation.

Variable	(1)	(2)	(3)	(4)	(5)	(6)	(7)	(8)
	L_dynSAR_1	L_dynSAR_2	L_dynSAR_3	L_dynSAR_4	H_dynSAR_1	H_dynSAR_2	H_dynSAR_3	H_dynSAR_4
lnphcb (β_1_)	−0.0348 ***	−0.0342 ***	−0.0336 ***	−0.0251 ***	−0.0301 ***	−0.0292 ***	−0.0278 ***	−0.0190 **
lrid (β_2_)	0.0092	0.0654			−0.1140 *	−0.0319		
lrid*lnphcb (β_3_)		−0.0616				−0.0899		
hrid (β_4_)			−0.0332	0.0648 *			−0.1059 **	−0.0017
hrid*lnphcb (β_5_)				−0.1056 ***				−0.1122 ***
Wy (ρ)	0.5027 ***	0.5030 ***	0.5018 ***	0.4990 ***	0.4849 ***	0.4857 ***	0.4793 ***	0.4747 ***
SR_Direct_lrid*lnphcb		−0.0635				−0.0933		
SR_Direct_hrid*lnphcb				−0.1117 ***				−0.1187 ***
SR_Indirect_lrid*lnphcb		−0.0607				−0.0808		
SR_Indirect_hrid*lnphcb				−0.1011 ***				−0.0990 ***
LR_Direct_lrid*lnphcb		−0.3208				−1.7027		
LR_Direct_hrid*lnphcb				−0.5676 ***				−0.6126
LR_Indirect_lrid*lnphcb		−0.0773				0.9411		
LR_Indirect_hrid*lnphcb				−0.0946				−0.3591

*Notes:* All control variables are included but not reported. * *p* < 0.10, ** *p* < 0.05, *** *p* < 0.01. *Source:* Authors’ original calculations using Stata.

**Table 6:** Further Analysis of heterogeneous food price risk mediation.

Variable	(1)	(2)	(3)	(4)	(5)	(6)	(7)	(8)	(9)	(10)	(11)	(12)
	lnlrfp_L	lnphcb_L	lnlrfp_L	lnlrfp_H	lnphcb_H	lnlrfp_H	lnhrfp_L	lnphcb_L	lnhrfp_L	lnhrfp_H	lnphcb_H	lnhrfp_H
lrid (β_1_)	−0.0097	0.5345 ***	0.0092				−0.1304 **	0.5383 ***	−0.1140 *			
hrid (β_2_)				−0.0473	0.2655 ***	−0.0332				−0.1173 **	0.2713 ***	−0.1059 **
lnphcb (β_3_)			−0.0348 ***			−0.0336 ***			−0.0301 ***			−0.0278 ***
Wy (ρ)	0.5151 ***	0.4723 ***	0.5027 ***	0.5134 ***	0.4652 ***	0.5018 ***	0.4946 ***	0.4716 ***	0.4849 ***	0.4876 ***	0.4644 ***	0.4793 ***
SR_Direct_lrid	−0.0050	0.5758 ***	0.0097				−0.1306 **	0.5795 ***	−0.1215 *			
SR_Direct_hrid				−0.0469	0.2844 ***	−0.0353				−0.1191 **	0.2905 ***	−0.1131 **
SR_Direct_lnphcb			−0.0361 ***			−0.0347 ***			−0.0308 ***			−0.0283 ***
SR_Indirect_lrid	−0.0041	0.4703 ***	0.0100				−0.1186 *	0.4731 ***	−0.1059			
SR_Indirect_hrid				−0.0441	0.2276 ***	−0.0312				−0.1075 **	0.2322 ***	−0.0985 **
SR_Indirect_lnphcb			−0.0331 ***			−0.0317 ***			−0.0267 ***			−0.0238 ***
LR_Direct_lrid	−0.0251	0.7182 ***	0.0491				−1.7689	0.7196 ***	−0.9735			
LR_Direct_hrid				−0.2379	0.3511 ***	−0.1788				−1.6648	0.3568 ***	−1.0412
LR_Direct_lnphcb			−0.1826 ***			−0.1762 ***			−0.3998			−0.3014
LR_Indirect_lrid	0.0035	1.0511 ***	0.0184				−0.7855	1.0281 ***	0.0892			
LR_Indirect_hrid				−0.0356	0.4871 ***	−0.0188				0.6168	0.4818 ***	0.1334
LR_Indirect_lnphcb			−0.0299			−0.0297			0.1676			0.0861
ME									−0.0162			−0.0075
MR									0.1245			0.0644

*Notes:* All control variables are included but not reported. * *p* < 0.10, ** *p* < 0.05, *** *p* < 0.01. *Source:* Authors’ original calculations using Stata.

## References

[1] Liu P., Ma L. (2016). Food scandals, media exposure, and citizens’ safety concerns: A multilevel analysis across Chinese cities. Food Policy.

[2] Barbarossa C., De Pelsmacker P., Moons I., Marcati A. (2016). The influence of country-of-origin stereotypes on consumer responses to food safety scandals: The case of the horsemeat adulteration. Food Qual. Prefer..

[3] Giacomarra M., Galati A., Crescimanno M., Tinervia S. (2016). The integration of quality and safety concerns in the wine industry: The role of third-party voluntary certifications. J. Clean. Prod..

[4] Kostkova P., Brewer H., de Lusignan S., Fottrell E., Goldacre B., Hart G., Koczan P., Knight P., Marsolier C., McKendry R.A. (2016). Who Owns the Data? Open Data for Healthcare. Front. Public Health.

[5] Yi L., Tao J.P., Tan C.F., Zhu Z.K. (2019). Avian Influenza, Public Opinion, and Risk Spillover: Measurement, Theory, and Evidence from China’s Broiler Market. Sustainability.

[6] Whitworth E., Druckman A., Woodward A. (2017). Food scares: A comprehensive categorisation. Br. Food J..

[7] Headey D.D., Martin W.J., Rausser G.C. (2016). The Impact of Food Prices on Poverty and Food Security. Annual Review of Resource Economics.

[8] Colchero M.A., Salgado J.C., Unar-Munguia M., Hernandez-Avila M., Rivera-Dommarco J.A. (2015). Price elasticity of the demand for sugar sweetened beverages and soft drinks in Mexico. Econ. Hum. Biol..

[9] Zhou J., Xu Y., Li C. (2019). The empirical analysis of the influencing mechanism of food safety incidents on the price fluctuations of livestock and poultry products. Res. Agric. Mod..

[10] Neuman W.R., Guggenheim L., Jang S.M., Bae S.Y. (2014). The Dynamics of Public Attention: Agenda-Setting Theory Meets Big Data. J. Commun..

[11] Lloyd T., McCorriston S., Morgan C.W., Rayner A.J. (2001). The impact of food scares on price adjustment in the UK beef market. Agric. Econ..

[12] Cai X., Tao J. (2017). The price fluctuation and its dynamic relations of the poultry industry chain under the influence of avian influenza. Res. Agric. Mod..

[13] Lucht J.M. (2015). Public Acceptance of Plant Biotechnology and GM Crops. Viruses.

[14] Zhou L., Turvey C.G., Hu W.Y., Ying R.Y. (2016). Fear and trust: How risk perceptions of avian influenza affect Chinese consumers’ demand for chicken. China Econ. Rev..

[15] Chen M.F. (2017). Modeling an extended theory of planned behavior model to predict intention to take precautions to avoid consuming food with additives. Food. Qual. Prefer..

[16] Serra T. (2015). Price volatility in Niger millet markets. Agric. Econ..

[17] Wang L.F., Duan W.J., Qu D., Wang S.J. (2018). What matters for global food price volatility?. Empir. Econ..

[18] Serra T., Zilberman D. (2013). Biofuel-related price transmission literature: A review. Energy Econ..

[19] Dillon B.M., Barrett C.B. (2016). Global Oil Prices and Local Food Prices: Evidence from East Africa. Am. J. Agric. Econ..

[20] Iregui A.M., Otero J. (2017). Testing for spatial market integration: Evidence for Colombia using a pairwise approach. Agric. Econ..

[21] Distefano T., Chiarotti G., Laio F., Ridolfi L. (2019). Spatial Distribution of the International Food Prices: Unexpected Heterogeneity and Randomness. Ecol. Econ..

[22] Hassouneh I., Radwan A., Serra T., Gil J.M. (2012). Food scare crises and developing countries: The impact of avian influenza on vertical price transmission in the Egyptian poultry sector. Food Policy.

[23] Camoglu S.M., Serra T., Gil J.M. (2015). Vertical price transmission in the Turkish poultry market: The avian influenza crisis. Appl. Econ..

[24] Hong H., Stein J.C. (1999). A Unified Theory of Underreaction, Momentum Trading, and Overreaction in Asset Markets. J. Financ..

[25] Li J.L., He C.Y., Liao D., He M.Y. (2018). Opinion Leadership, Limited Attention and Overreaction. Econ. Res. J..

[26] Kahneman D. (1973). Attention and Effort.

[27] Lazarsfeld P.F., Berelson B., Gaudet H. (1944). The People’s Choice: How the Voter Makes up His Mind in a Presidential Campaign.

[28] Aboody D., Lehavy R., Trueman B. (2010). Limited attention and the earnings announcement returns of past stock market winners. Rev. Account. Stud..

[29] Wang X.Q., Weldegebriel H.T., Rayner A.J. (2007). Price Transmission in Vertically Related Markets. China Econ. Q..

[30] Dyl E.A., Yuksel H.Z., Zaynutdinova G.R. (2019). Price reversals and price continuations following large price movements. J. Bus. Res..

[31] Getis A., Aldstadt J. (2004). Constructing the Spatial Weights Matrix Using a Local Statistic. Geogr. Anal..

[32] Yu Y.Z., Xuan Y., Shen Y.Y. (2013). The Spatial Spillover Effect and Region Boundary of Financial Agglomeration on Industrial Productivity: The Spatial Econometric Research of 230 Cities. J. World Econ..

[33] Baron R.M., Kenny D.A. (1986). The moderator-mediator variable distinction in social psychological research: Conceptual, strategic, and statistical considerations. J. Personal. Soc. Psychol..

[34] Belotti F., Hughes G., Mortari A.P. (2017). Spatial panel-data models using Stata. Stata J..

[35] Wooldridge J.M. (2016). Introductory Econometrics: A Modern Approach.

[36] Hayes A.F. (2017). Introduction to Mediation, Moderation, and Conditional Process Analysis: A Regression-Based Approach.

[37] Mackinnon D.P., Warsi G., Dwyer J.H. (1995). A Simulation Study of Mediated Effect Measures. Multivar. Behav. Res..

[38] Liu Y., Liu F.Y., Zhang J.F., Gao J.B. (2015). Insights into the nature of food safety issues in Beijing through content analysis of an Internet database of food safety incidents in China. Food Control.

[39] Luo J., Wang J.P., Zhao Y.L., Chen T.Q. (2018). Scare Behavior Diffusion Model of Health Food Safety Based on Complex Network. Complexity.

[40] Byerlee D., Jayne T.S., Myers R.J. (2006). Managing food price risks and instability in a liberalizing market environment: Overview and policy options. Food Policy.

[41] Renn O. (2005). Risk perception and communication: Lessons for the food and food packaging industry. Food Addit. Contam..

[42] Dodson C.S., Holland P.W., Shimamura A.P. (1998). On the recollection of specific- and partial-source information. J. Exp. Psychol. Learn. Mem. Cognit..

[43] Cacciatore M.A., Anderson A.A., Choi D.H., Brossard D., Scheufele D.A., Liang X., Ladwig P.J., Xenos M., Dudo A. (2012). Coverage of emerging technologies: A comparison between print and online media. New Media Soc..

[44] Lin H.C., Bruning P.F., Swarna H. (2018). Using online opinion leaders to promote the hedonic and utilitarian value of products and services. Bus. Horiz..

[45] Omari R., Frempong G.K., Arthur W. (2018). Public perceptions and worry about food safety hazards and risks in Ghana. Food Control.

[46] Shan L.J., Wu L.H., Xu L.L. (2012). An empirical study on consumer perception of food safety risk—An example of food additives. J. Food Agric. Environ..

[47] Hendershott T., Menkveld A.J. (2014). Price pressures. J. Financ. Econ..

[48] Zhang B., Wang Y. (2015). Limited attention of individual investors and stock performance: Evidence from the ChiNext market. Econ. Model..

[49] Yao T., Zhang Y.J., Ma C.Q. (2017). How does investor attention affect international crude oil prices?. Appl. Energy.

[50] Han J.Q., Xu L. (2017). Spatial econometric analysis of price fluctuation of Broiler in China. Price Theory Pract..

[51] Apergis N., Rezitis A. (2003). Agricultural price volatility spillover effects: The case of Greece. Eur. Rev. Agric. Econ..

[52] Von Cramon-Taubadel S. (1998). Estimating asymmetric price transmission with the error correction representation: An application to the German pork market. Eur. Rev. Agric. Econ..

[53] Baulch B. (1997). Transfer costs, spatial arbitrage, and testing for food market integration. Am. J. Agric. Econ..

[54] Mainardi S. (2011). Cropland use, yields, and droughts: Spatial data modeling for Burkina Faso and Niger. Agric. Econ..

[55] Lee J.H., Choi Y.J. (2009). News values of sports events: An application of a newsworthiness model on the World Cup coverage of US and Korean media. Asian J. Commun..

[56] Miles S., Frewer L.J. (2001). Investigating specific concerns about different food hazards. Food. Qual. Prefer..

[57] Zhang H., Gao N., Wang Y., Han Y.X. (2018). Modeling risk governance and risk perception in personal prevention with regard to food safety issues. Br. Food J..

[58] Prentice C., Chen J., Wang X.Q. (2019). The influence of product and personal attributes on organic food marketing. J. Retail. Consum. Serv..

[59] Berg L., Gornitzka A. (2012). The consumer attention deficit syndrome: Consumer choices in complex markets. Acta Sociol..

[60] Weber B. (2014). Neuroscience Consumer Behavior Research—Where the Brain Research can help?. J. Verbrauch. Lebensm..

[61] Yu H.Y., Sirsat S.A., Neal J.A. (2019). Linking food safety training with whistle-blowing: The mediation roles of job satisfaction and self-efficacy. Int. J. Contemp. Hosp. Manag..

[62] Sanlier N., Baser F. (2019). The Relationship among Food Safety Knowledge, Attitude, and Behavior of Young Turkish Women. J. Am. Coll. Nutr..

